# Supplementary material for: Bionitio: demonstrating and facilitating best practices for bioinformatics command-line software
Source: Gigascience. 2019 Sep 23;8(9):giz109. doi: 10.1093/gigascience/giz109 (PMC6755254; doi:10.1093/gigascience/giz109)
Supplement: giz109_GIGA-D-19-00145_Revision_1 [file giz109_giga-d-19-00145_revision_1.pdf]

GigaScience

Bionitio: demonstrating and facilitating best practices for bioinformatics command-line software

--Manuscript Draft--

**Manuscript Number:**	GIGA-D-19-00145R1
**Full Title:**	Bionitio: demonstrating and facilitating best practices for bioinformatics command-line software
**Article Type:**	Technical Note
**Funding Information:**	
**Abstract:**	Background<br><br>Bioinformatics software tools are often created ad hoc, frequently by people without extensive training in software development. In particular, for beginners, the barrier to entry in bioinformatics software development is high, especially if they want to adopt good programming practices. Even experienced developers do not always follow best practices in all the code they develop. A consequence of this is the proliferation of poorer-quality bioinformatics software, leading to limited scalability and inefficient use of resources; lack of reproducibility, usability, adaptability and interoperability; and erroneous or inaccurate results.<br><br>Findings<br><br>In response to this problem we have developed Bionitio, a tool that automates the process of starting new bioinformatics software projects following recommended best-practices. With a single command, the user can create a new well-structured project in one of twelve programming languages. The resulting software is functional, carrying out a prototypical bioinformatics task, and thus serves as both a working example and a template for building new tools. Key features include command line argument parsing, error handling, progress logging, defined exit status values, a test suite, a version number, standardised building and packaging, user documentation, code documentation, a standard open-source software license, software revision control, and containerisation.<br><br>Conclusions<br><br>Bionitio serves as a learning aid for beginner-to-intermediate bioinformatics programmers and provides an excellent starting point for new projects. This helps developers adopt good programming practices from the beginning of a project and encourages high-quality tools to be developed more rapidly. This also benefits users of the tools because they are more easily installed and consistent in their usage. Bionitio is released as open source software under the MIT License, and is available at https://github.com/bionitio-team/bionitio.
**Corresponding Author:**	Bernie Pope, Ph.D.<br>The University of Melbourne, Australia<br>AUSTRALIA
**Corresponding Author Secondary Information:**	
**Corresponding Author's Institution:**	The University of Melbourne, Australia
**Corresponding Author's Secondary Institution:**	
**First Author:**	Peter Georgeson
**First Author Secondary Information:**	
**Order of Authors:**	Peter Georgeson
	Anna Syme

Powered by Editorial Manager® and ProduXion Manager® from Aries Systems Corporation

	Clare Sloggett
	Jessica Chung
	Harriet Dashnow
	Michael Milton
	Andrew Lonsdale
	David Powell
	Torsten Seemann
	Bernard Pope, Ph.D.
**Order of Authors Secondary Information:**	
**Response to Reviewers:**	16 July 2019<br><br>To the Editor-in-Chief and Executive Editor, GigaScience,<br><br>We thank the Editor and Reviewers for their insightful and constructive comments on our submission "Bionitio: demonstrating and facilitating best practices for bioinformatics command-line software" (GIGA-D-19-00145). In the following document we respond to each of the reviewer comments and say what has changed in the software and text to address each point. In the revised manuscript we have used red font to indicate changes that we have made to the body of the text. We believe that the suggested changes have significantly improved the quality of the paper and the corresponding Bionitio tool.<br><br>The responses here are presented in the order that the comments appear in the manuscript review.<br><br><br>Editor Comments<br><br>The reviewers agree that the tool itself is a useful contribution, overall. However, they also have some constructive suggestions for improving the manuscript.<br><br>In particular, I agree with reviewer 1 that, ideally, the manuscript should also present "an evidence-backed testimony about the tool's efficacy in correcting the problems stated in the introduction."<br>I understand that a typical, quantitative benchmarking exercise may not be possible for this type of tool, but reviewer 1 has some good pointers regarding issues that should be discussed in more detail (for example, regarding FAIR principles and how your approach suggested in the paper can help in this regard).<br><br>Reviewer 2 has some notes on installation and running the tool that may give you some hints for minor improvements or corrections.<br><br>The reviewers also suggest to provide the tool via a container (e.g. docker), especially as it is meant to be helpful for beginners.<br><br>In addition, please register any new software application in the SciCrunch.org database to receive a RRID (Research Resource Identification Initiative ID) number, and include this in your manuscript. This will facilitate tracking, reproducibility and re-use of your tool.<br><br>Response<br><br>We concur with the Editor that a qualitative benchmarking exercise is challenging for this type of tool, and that a detailed discussion of our alignment with FAIR principles is a valuable contribution to the paper. In light of these remarks we have included a section on how Bionitio enables bioinformaticians to easily adopt many of the key FAIR principles and have additionally linked this to recent work on related recommendations for Open Source research software. Disclaimer: a Bionitio author (B Pope) a co-author on the latter recommendations.

Powered by Editorial Manager® and ProduXion Manager® from Aries Systems Corporation

We have attempted to address issues related to installation and running the tool outlined by Reviewer 2 and have provided Docker containers for each of the Bionitio implementations, as well as the bootstrap script, and updated the documentation accordingly.

We have applied for registration of Bionitio through SciCrunch.org and received an RRID of SCR_017259. We have included this in the manuscript.

Reviewer 1

Comment 1 (and comment 12)

The limitation of this manuscript, in my mind, is mostly that it reads like more of an instruction manual and list of general best practices than a detailed technical write up about the contribution made, and an evidence-backed testimony about its efficacy in correcting the problems stated in the introduction.

Response

We believe that one of the contributions of Bionitio is that it provides a consolidation of many disparate sources of best practices for software development in bioinformatics. Indeed, the features present in Bionitio are distilled from more than 25 different partially overlapping recommendations. We also believe that it is a contribution of our manuscript to explicitly link those recommendations to the features present in our tool. Therefore, there is necessity to list our sources and argue for their significance. Another key contribution of our paper is to show how easily a new project can be created with our tool, as a step-by-step guide to its main features.

However, we also agree that our manuscript could have made our contributions clearer and argued further for its efficacy in correcting the problems stated in the introduction.

We also agree with the Editor that "a typical, quantitative benchmarking exercise may not be possible for this type of tool".

In light of these comments we have made considerable changes to the manuscript.

The following text was added to the conclusion to show how Bionitio helps users to adopt FAIR principles and related recommendations for open source software:

Alignment with FAIR Principles and OSS Recommendations

In an effort to facilitate continued benefit from the digital assets related to data-intensive science, representatives from academia, industry, funding agencies, and publishers have proposed the FAIR Data Principles that aim to make experimental artefacts findable, accessible, interoperable and reusable for machines and people [48]. Jiménez et al have argued that poor development practices result in lower quality outputs that negatively impact reproducibility and reusability of research [49], and propose four principles for open source software development (OSS recommendations) that align well with the FAIR principles: 1) make source code publicly accessible from day one; 2) make software easy to discover by providing software metadata via a popular community registry; 3) adopt a licence and comply with the licence of third-party dependencies; and 4) define clear and transparent contribution, governance and communication processes. Tools developed with Bionitio have a head start on satisfying both the FAIR principles and the first three OSS recommendations:

- they are publicly accessible in GitHub repositories with clearly indicated standard open source licences and user documentation;
- they are interoperable with other tools via standardised inputs and outputs and interfaces that follow long-established conventions;
- they are re-usable by virtue of the adoption of standard build procedures, the provision of clear documentation relating to installation and usage, containerisation with Docker, and integration into CWL;
- where appropriate, specific versions (with defined version numbers) can be made

findable by the allocation of Digital Object Identifiers facilitated by Zenodo [50] through GitHub.
Importantly, Bionitio facilitates compliance with these principles, which is seen by Jiménez et al as the final (and, in our opinion, most difficult) step in organisational adoption.

The following text was added to the conclusion to outline Bionitio's role in education and training by relating it to the Mastery Rubric for Bioinformatics proposed by Tractenberg et al, along with our own experience in using it to deliver a national bioinformatics workshop (in Australia):

Role in education and training

In very recent work Tractenberg et al have developed a Mastery Rubric for Bioinformatics with the goal of better defining skills development and competencies in the discipline [46]. In this framework, competency in computational methods ranges through five levels, from novice (stage 1) to independent bioinformatics practitioner (stage 5). One of the goals of Bionitio is to support education and training for advancing bioinformaticians from stage 3 - learning best practices in programming and writing basic code - to stage 5 - developing new software that is useful, efficient, standardized, well-documented and reproducible. As an example of this application, Bionitio was used as the basis for a whole-day workshop on best practices in bioinformatics software development at the Australian Bioinformatics and Computational Biology Society (ABACBS) Annual Conference in November 2018 [47], delivered to an audience of 50 bioinformaticians from research and clinical institutes around Australia. In the first half of the workshop participants learnt how to set up a new software repository using Bionitio, allowing time for exploration of the codebase, discussion of key aspects of quality software, and an explanation of the processes that are automated by Bionitio. In the second half of the workshop participants learnt about test-driven development (TDD) and undertook an exercise to extend the codebase with new features, documentation, corresponding test cases, and linkage to revision control and continuous integration testing. In this setting, Bionitio's design as a simple-yet-realistic bioinformatics exemplar provides both a common codebase for coordination of workshop materials and an extensible platform for the delivery of hands-on practical activities. Additionally, by providing complete working examples in many different languages, Bionitio acts as a kind of "Rosetta Stone" and is therefore likely an excellent vehicle for comparative programming skills transfer.

We have also expanded the third paragraph in the Conclusions to emphasise why we think Bionitio is a significant contribution on top of the already existing recommendations in the literature (and the main motivation for its creation):

The challenges faced by the bioinformatics and science communities in building better quality software are well known, and there is no shortage of practical recommendations to be found in the literature. These guidelines are undoubtedly useful to beginners, however we believe they fall short in two ways. First, they are spread over multiple manuscripts that only partially overlap in their recommendations, therefore some level of consolidation is needed. Second, they are static artefacts that point to good practices but do not remove the considerable burden of applying them in real code. These two observations motivated the creation of Bionitio, both as a way of collecting commonly recommended best practices, and as a way of demonstrating and facilitating their use. Therefore, a significant contribution of our work is to build a tool that can both illustrate best practices by example but also make it easy to use them in new projects. In this sense Bionitio takes a much more active role in the dissemination and compliance with these principles.

We have also emphasised the contribution that this tool makes to improving software development in bioinformatics as per comments 13,14,15 and 21 below.

Comment 2

(section 1; paragraph 2) How is "correctness" evaluated in your mind? In research truth is often unknown by definition, so perhaps choose a less loaded word or elaborate on how this is evaluated.

Powered by Editorial Manager® and ProduXion Manager® from Aries Systems Corporation

Response

We agree with the reviewer that truth can be elusive in science, and therefore by correctness we mean that the software implements its intended functionality; so it is correct in the sense that it meets its specification (whether that specification be formally defined, or, more likely, part of the informal intentions that are known to the author(s)). Following the advice of the reviewer we have used a less loaded way to describe this, and changed the manuscript as follows:

Given the results-driven nature of research, the functional aspects of scientific programs (e.g. correctness whether expected inputs produce expected outputs) are heavily emphasised at the expense of the non-functional ones (e.g. usability, maintainability, interoperability, efficiency).

Comment 3

Duplicate heading at start of paper? Both "Findings" and "Background"

Response

We believe that this formatting follows the suggested GigaScience Technical Note style (https://academic.oup.com/gigascience/pages/technical_note), where in the main text, "Findings" is a larger heading including the subheadings Background, Implementation, Methods, Conclusions, etc. If our interpretation of the formatting guidelines is incorrect, we are confident that this can be fixed in the final proof.

Comment 4

(section 1; paragraph 2, last sentence) Some "specifications" or recommendations, such as Nature Publishing's software checklist, and some 10-simple-rules articles in pnas related to scientific software. Are these the types of things you're referring to? If so, might be worth mentioning how they can exist but perhaps are harder to define for a specific (quickly moving) domain beyond the "basics".

Response

In this part we are referring to "software requirements specifications" that are commonly used in Software Engineering to define the functional and non-functional requirements of software being developed. We have changed the text to "software requirements specifications (SRSs)" to clarify this point.

Comment 5

(section 1; paragraph 4) abovementioned -> above-mentioned

Response

Corrected.

Comment 6

(section 1; second-last paragraph) "more likely to adopt good practices" <- have you witnessed this in the wild with bionitio, yet? I agree that in principle I'd expect this result, but giving students or researchers the tool and saying nothing else, then coming back at the end of the process, is this the outcome we get? The biggest places I see this not continuing beyond the boilerplate is documentation and testing. This could potentially also be answered if Cookiecutter has successes that you could reference.

Response

We agree with the reviewer that this is an expected result, however we have not formally tested it, and, for now it sits here as a hypothesis. We have reworded the sentence to make this point clearer:

The key point is that they are building on solid foundations, and because a lot of the mundane-but-important boilerplate is provided by Bionitio, there are fewer barriers to adopting good practices from the start.

Comment 7

(command line argument parsing) have you considered integrating these command-line descriptions with standard tools for shipping workflows to C(G)PUs, like Common Workflow Language (commonwl.org), Boutiques (boutiques.github.io), or others? It would be an additional feature you could add on top of each language-specific implementation that would make not only consuming the tools even more uniform, but enable scaling them out for large datasets more accessible for developers.

Response

We agree with the reviewer that this would be a useful additional feature, and therefore have added example CWL tool wrappers for each implementation of Bionitio. This addition was greatly facilitated by the fact that each Bionitio implementation has the same command line interface, and (now) comes with a Docker container. We have updated the online documentation for Bionitio to include information about this, and have made the following changes to the manuscript:

In the Background section:

Operating system virtualisation services, such as Docker [22], and workflow specification languages, such as the Common Workflow Language (CWL) [23], have improved portability and reproducibility of tools and pipelines [12,24–26].
...

Specifically, every new Bionitio-created project includes … containerisation with Docker, and a CWL wrapper.

In the Design and Implementation section:

CWL tool wrapper

Bioinformatics pipelines — where multiple tools are chained together to perform an overall analysis — create further challenges for reproducible science. This has motivated the creation of pipeline frameworks that allow the logic of such computations to be abstracted from the details of how they are executed. An emerging standard in this area is the Common Workflow Language (CWL) that is supported by several popular workflow engines. CWL comprises two declarative sub-languages: workflow descriptions, that define data flow patterns between pipeline stages; and command line tool descriptions, that define the interfaces of tools in a platform independent manner. Each Bionitio implementation provides a CWL tool description "bionitio.cwl", that facilitates its incorporation into CWL pipelines, and takes advantage of CWL's support for invoking programs within Docker containers.

We have also updated the README.md files for each implementation of Bionitio to include information about how to use the CWL tool wrapper and included running the CWL tool wrapper within Travis CI testing.

Comment 8

(software packaging) there is also no mention of virtualization/containerization here, such as Docker or Singularity, that would also increase the portability of these packages. Have the authors considered this to further minimize this issue?

Response

We agree with the reviewer that this would be a useful additional feature, and therefore have added example Docker container definitions for each implementation of Bionitio,

and also the bootstrap script.
We have made the following changes to the manuscript:

In the Abstract:

Key features include … , and containerisation.

In the Background section:

Specifically, every new Bionitio-created project includes … containerisation with Docker, and a CWL wrapper.

In the Design and Implementation section:

Sub-heading changed from "Standardised software packaging using programming language specific mechanisms" to "Standardised software packaging and containerisation".

Text added:

Standard packaging also helps with containerisation, which is becoming increasingly useful in bioinformatics [40]. Docker containers are a popular implementation of this concept, where the underlying operating system is virtualised and packaged alongside tools and their dependencies. This makes it easy to install "containerised" software on any platform that supports Docker, and facilitates reproducibility by enabling the exact same software build to be used on every system. Each Bionitio implementation comes with a "Dockerfile" that encodes all the necessary information needed to create a containerised version of the tool. As an added benefit, the Docker container is used in Travis Continuous Integration testing, which both simplifies the use of Travis and also enables the functionality of the container itself to be included in the tests.

In the Methods section we added the following text:

Alternatively, the bootstrap script can be run from a Docker container published on DockerHub (https://cloud.docker.com/u/bionitio/repository/docker/bionitio/bionitio-boot):

```
$ docker run -it -v "$(pwd):/out" --rm bionitio/bionitio-boot \
-i python -n newproj -c BSD-3-Clause
```

Comment 9

(methods; choosing a language) do you have any way to recommend language selection for users? If they're truly new to all of these, maybe coming from a MATLAB background like many who learned to program through coursework, what guidance does Bionitio provide here? Is Python a general default, or just for this example? If it is, where is that justified? The caveat with providing 12 options is that a bit of hand holding may be required to guide the choice for much of your target audience.

Response

We agree with the reviewer that choice of programming language can be difficult for absolute beginners. It is difficult to get empirical evidence to support any language default (and for this reason Bionitio does not have a default language). However, the selection of implementation languages chosen was guided by the results reported in [13]. From an analysis of 1,720 bioinformatics repositories on GitHub they observed: "The main dataset contained a greater proportion of code written in interpreted or hybrid interpreted/compiled (such as Python) and dynamically typed languages" and "Our data support the intuition that Java, Python and R are more succinct than lower-level languages such as C and C++"
Taking these observations together, Python appears to be reasonable starting language for beginners. To assist beginners with their choice of language we have updated the README (https://github.com/bionitio-team/bionitio) documentation for Bionitio to include:

If you are new to programming, and do not know which programming language to use, then we recommend picking one of the high-level interpreted languages that are popular in Bioinformatics, such as Python or R. You may also need to seek advice from your peers about which language(s) are most appropriate for your purposes. We have tried to cover as many popular languages as possible, and apologise if your preference is not currently available. However, we also welcome new implementations of Bionitio in languages not already covered.

We have also added the following text to the manuscript:

For users relatively new to programming, with no prior constraints on their choice of language, we recommend they choose a high-level interpreted language such as Python or R.

Comment 10

can you justify the claims about it being an "excellent vehicle for education"? Any sort of case study or example from similar tools being effective, etc…

Response

We believe that Bionitio is fairly unique in its approach to templating best practices in Bioinformatics software development, and therefore it is unlikely that such an approach has been formally studied in the context of education practices, and unfortunately we are not aware of such resources (even beyond bioinformatics). However, as mentioned in our cover letter, we have used Bionitio as the basis for a popular (whole day) workshop hosted at the Australian Bioinformatics and Computational Biology Society (ABACBS) annual conference in 2018 (https://www.abacbs.org/conference2018) with ~50 paying attendees from around the country. We conducted a survey of the attendees to assess the quality and utility of the workshop. In response to the question "This was a useful workshop that enhanced my knowledge and skills" out of 18 respondents 94.44% agreed or strongly agreed. Given the success of the initial workshop, we ran another in May 2019, with 14 attendees. From formal feedback received from the second workshop, in response to the question "My overall impression is that this is a useful workshop that enhanced my knowledge and skills" we received a score of 4.8/5 from 11 respondents. We appreciate that this is anecdotal evidence and is not supported by a rigorous experiment and therefore we have not discussed the workshop feedback in the manuscript. However, we have reduced the strength of our claim in the manuscript by adding a qualifier:

Additionally, by providing complete working examples in many different languages, Bionitio acts as a kind of "Rosetta Stone" and is therefore likely an excellent vehicle for comparative programming skills transfer.

We have also addressed Bionitio's role in training and education more thoroughly in the Conclusion as mentioned in our response to Comment 1 above.

Comment 11

figure 1 text is barely readable, and boxes are odd relative sizes with a fair amount of wasted foreground (coloured) space. Colour doesn't seem to convey much information. I didn't find this figure particularly useful or instructive. I.e. I don't know any better how I would use bionitio, or what exactly it'll create (just that it draws from a boiler plate). Maybe repurpose this figure to be more of a "schematic" of what is contained within a bionitio-created-project (is there a more concise name for these?), and then a more streamlined version of what is currently here.

Response

We included this figure in the manuscript because it serves to visually represent how a new project is started by the bootstrap script. We have found that this has been a particular issue of confusion for new users, especially those who are unfamiliar with Git

Powered by Editorial Manager® and ProduXion Manager® from Aries Systems Corporation

and GitHub. The colours represent the location of code, either in GitHub (yellow) or the local machine (purple). To streamline this figure, we have removed the grey background, replaced the external arrows with dashed lines. We will submit a high-resolution version in our resubmission, and include a resolution-independent SVG version as well.

Comment 12

I felt that while the manuscript introduces a tool which is certainly of use to a community of scientific software developers, the focus of the paper is more based on the justification of which components are included in this tool, rather than the technical nature or efficacy of the contribution. With guides that exist and "best practices" that were even mentioned in the 10-simple-rules article, I believe the article would benefit from significant rewriting to be focused on the contributions of these authors and their tool, rather than an extended summary of what are commonly accepted as best practices for software development. While I acknowledge the novel and valuable contribution presented in this paper, I feel the manuscript does not highlight this contribution adequately.

Response

Thank you for recognising the utility of Bionitio. We believe this point is similar in to Comment 1 which we have addressed in detail above.

Comment 13

One concern I have with making it easier for people to continue making their own tools is exactly to the point mentioned at one point in the manuscript, of "never repeat yourself." In my area of research, computational neuroscience, essentially every pipeline has been built handfuls of times, and the answers aren't particularly replicable across implementations. This of course raises a whole other set of issues in terms of the quality of software being produced, because even if we encourage developers to adopt existing tools where possible, if they don't adopt the same ones for the same tasks, how can we meaningfully compare their implementations? This ties in to the FAIR principles, which I was surprised not to see mention of in this work, as they are closely aligned with the aim of bionitio to my understanding. The missing piece in bionitio, of course, would then be that of publishing tools and ensuring the findability of software that people will make. This of course doesn't solve the issue, but at least enables the easier evaluation of various implementations towards the same end. As mentioned above, the Boutiques initiative (disclaimer: I am a co-lead on this project) makes efforts to make sure tools, once they exist, are able to be shared/consumed FAIR-ly, so could potentially be referenced in a discussion on this point. The paper of this tool is on Gigascience (https://doi.org/10.1093/gigascience/giy016) and a recent poster focusing on FAIR software workflows can be found here: https://doi.org/10.6084/m9.figshare.8143241.v2 . I would appreciate if the authors discussed this point, the obvious risk that their tool introduces into the field by virtue of increasing the accessibility of tool development, and how they propose their contribution is either worth this added risk or how they intend on enabling the evaluation of it.

Response

We agree with the reviewer that there can be a tendency in bioinformatics and scientific computing to reinvent the wheel, which can create challenges for replication and comparison. However, a key point is that the purpose of Bionitio is not to merely make it easier for people to continue making their own tools or make software development more accessible (though many other systems, such as IDEs, do this already), but rather to help people to make better tools. We anticipate that many uses of Bionitio will be for bespoke tools that are tailored to the specific needs of a research project - tools that do not already exist. In our experience there is considerable demand in Bioinformatics for the creation of this type of software. Even if these tools do not have a wide audience at the beginning, we still believe that there is considerable value to be gained from the use of good programming practices from the outset of the project, as discussed in the paper. We also agree with the reviewer that the FAIR

Powered by Editorial Manager® and ProduXion Manager® from Aries Systems Corporation

principles are highly relevant and have made considerable changes to the manuscript to address this issue, as per our response to Comment 1. We thank the reviewer for bringing the Boutiques system to our attention. Command-line Interface and packaging standardisation efforts provided by Boutiques and similar systems such as CWL provide great utility in building usable and reproducible analyses. We commend and support these initiatives. We have added CWL tool wrapper support to Bionitio as discussed in our response to Comment 7 above.

Reviewer 2

Comment 14

I wonder if the command line is the best starting point for inexperienced programmers? And if not starting with an IDE that provides similar templates would be better? The paper could therefore discuss how bionitio can be used from an IDE. I also wonder if the team plan to provide bionitio templates for some popular IDEs?

Response

We agree that providing support for IDE use may be of use to inexperienced programmers, however that would be challenging to do in a way that is portable across: all the (12) language implementations of Bionitio, the many popular IDEs that are currently in use, and the major operating systems. Considering these challenges, we decided to make Bionitio agnostic with respect to the programming environment employed by users. Users can use an IDE with Bionitio if they desire, but we do not plan to offer particular support for any of the many options available.

Comment 15

are there some numbers that can document how much bionitio is used, and who the users are? Perhaps something from GitHub?

Response

We do not currently have information about how much Bionitio is used and who the users are. We use it ourselves on a regular basis, as do many of our colleagues. Many attendees of our workshops continue to use it for their own work. Our experience shows that it offers considerable utility for a wide audience.

Comment 16

I tested bionitio-python in Ubuntu on Windows. It has Python2 as default, so by following the instructions I could install the code (with Python2), but not run it. However, by installing the code with pip3 I could use it as described in the documentation. But this solution may not be obvious for beginner programmers, so the documentation should take this into account.

Response

Only Python 3 is currently supported by Bionitio. Python 3 is at least 10 years old, and Python 2 is now officially considered a legacy system. New Python projects are encouraged to be written in Python 3. As the reviewer notes, it is unfortunate that some operating systems still offer Python 2 as a default. As suggested, we have updated the user documentation to make it clearer that Python 3 is required. Specifically, we have replaced Python with "Python 3" on the main README.md for Bionitio (https://github.com/bionitio-team/bionitio), and also added the text "Python 3 is required for this software." on the README.md for the Python implementation of Bionitio (https://github.com/bionitio-team/bionitio-python).

Comment 17

I also found that the example program treated invalid fasta files (for example setup.py) as empty fasta files. I believe according to the documentation this should have resulted

in an "invalid file" exit value instead of success (zero).

Response

We thank the reviewer for identifying this mistake. Most FASTA parsing libraries are very liberal in what they accept as valid input, and therefore do not raise errors on files that are not FASTA format. We had originally intended to return an exit status of 3 for input files not in FASTA format, but this is challenging to do when most standard libraries for parsing FASTA do not raise errors. Given this limitation, we have decided to remove the exit status of 3 and have adjusted the documentation in the README file for each Bionitio implementation accordingly.

Comment 18

In the documentation, C# was not mentioned as one of the available languages.
The integration with GitHub, however, did not work. When we ran the script, a different command-line window was opened, asking for a GitHub password. When typing in the password, it was shown in clear text. Nothing happened when hitting the Enter-key. The GitHub-repository was not created.

Response

We thank the reviewer for pointing out the omission of C# in the list of available languages. We have corrected this mistake in the README.md file for Bionitio (https://github.com/bionitio-team/bionitio/blob/master/README.md) and in the user documentation (https://github.com/bionitio-team/bionitio/wiki/2.-Set-up-a-project-with-bionitio).

We have not been able to reproduce the error with GitHub integration on Windows. However, we have created an issue on our GitHub repository for Bionitio to look into this further, to see if it can be reproduced (https://github.com/bionitio-team/bionitio/issues/73).

Comment 19

The C++ version requires a bit more manual configuration, since the `CMake`/`Make` install does not copy the built executable to a standard location (such as usr/local/bin). It also depends on the `seqan` library that has to be downloaded separately.

Response

We agree with the reviewer that the C++ version requires more manual configuration than some of the other implementations of Bionitio. This is due to the limited nature of C++ software packaging systems. We require the user to download the Seqan source code from http://packages.seqan.de/seqan-library/seqan-library-2.1.1.tar.xz because we do not want to include that source code in the Bionitio repository. We have added an 'install' target for the build command now, so that the user can run 'make install' and have the executable copied into their desired location and have updated the documentation accordingly (https://github.com/bionitio-team/bionitio-cpp/blob/master/README.md). The default behaviour is to place the executable in the same directory where the 'make' command was executed. It is also likely that the added support for Docker will also help some users with this issue.

Reviewer 3

Comment 20

for Windows computer use case scenario, it only say to get Putty to login to Linux. I would suggest the authors create a Virtualbox or Docker container (both virtualizations solutions are as easy to install on Windows as you would install Skype), with some base ubuntu and some working examples included of project structure generation with their tool.

	Response<br><br>Currently Bionitio is not directly supported on Windows. Our user guide suggested that Windows users might need to log into a Unix-like system to try it out. We have added the reviewer’s suggestion that they may also like to consider the use of a virtualisation system such as VirtualBox (https://github.com/bionitio-team/bionitio/wiki/1.-Set-up-your-computer). We agree with the reviewer that the addition of Docker container support will be a benefit to Windows users.<br><br>Comment 21<br><br>It would be great to have a little discussion how it can help beginner developers, for example can it be used in an educational setting when setting up projects for a bioinformatics class. It could be also discussed how advanced developers can use that to get project structure initiated fast (I am thinking Ruby on Rails or Django project initiation). What are the additional benefits for advanced developers ?<br><br>Response<br><br>Yes, Bionitio is well suited to being used in an educational setting. We have developed and delivered workshops on best practices in bioinformatics software development based on Bionitio. We have added a new section to the manuscript on its role in education and training (see our previous response to Comment 1 above). We agree that Bionitio can also help advanced users get project structure initiated quickly. Indeed, we count ourselves as advanced users, and we regularly use Bionitio for this purpose. We believe that the use-case suggested by the reviewer (Ruby on Rails or Django project) is better solved by the Cookiecutter project, mentioned as related work in the Background section where we note "Cookiecutter provides a more general-purpose templating system that is best suited to starting new software systems in specific programming languages, such as the instantiation of web applications based on particular web framework libraries."<br><br>Additional changes to the manuscript<br><br>The following small improvements were made to the manuscript.<br><br>1) Affiliation updated for one author:<br><br>Melbourne Genomics Health Alliance, Walter and Eliza Hall Institute, Parkville, Victoria, Australia.<br><br>2) A sentence in the Abstract was reworded to improve expression:<br><br>Old sentence:<br><br>On the other hand, the barrier to entry in bioinformatics software development is high for beginners, especially if they want to adopt good programming practices.<br><br>Replacement:<br><br>In particular, for beginners, the barrier to entry in bioinformatics software development is high, especially if they want to adopt good programming practices.<br><br>3) Text added to describe the optional --minlen command line argument in the Design and Implementation section:<br><br>An optional command line argument --minlen can be supplied, causing the program to ignore sequences with length strictly less than the given value.<br><br>4) Paragraph slightly reworded in the Design and Implementation section to improve expression:

	Old paragraph:<br><br>In the remainder of this section we outline the main features incorporated into Bionitio's prototypical tool that facilitate good programming practices and why they are important. In the following section we demonstrate by example how Bionitio can be used to create a new software project.<br><br>New paragraph with changes noted in red:<br><br>In the remainder of this section we outline the main features incorporated into Bionitio's prototypical tool that facilitate good programming practices, and, where possible, relate them to the relevant recommendations in the literature. In the section afterwards we demonstrate by example how Bionitio can be used to create a new software project.<br><br>5) Replaced "in name of" with "for the sake of" in the Conclusion to improve expression.<br><br>6) We capitalised "Cookiecutter" in the Background section.<br><br>7) We shifted the sentence "(the $ sign indicates the command line prompt):" earlier in the text, to cover the first use of the $ notation.<br><br>8) We improved the wording of the first sentence in the sub-section "Standardised software packaging and containerisation" (and added an additional reference): "The installation process can be one of the most cumbersome and frustrating parts of using bioinformatics software, because many tools do not provide much assistance to the user [14], and complex dependency chains can clash with local settings [10]."<br><br>9) Fixed the pluralisation in the Revision control sub-section: "implementations"
**Additional Information:**	
**Question**	**Response**
Are you submitting this manuscript to a special series or article collection?	No
**Experimental design and statistics**<br><br>Full details of the experimental design and statistical methods used should be given in the Methods section, as detailed in our Minimum Standards Reporting Checklist. Information essential to interpreting the data presented should be made available in the figure legends.<br><br>Have you included all the information requested in your manuscript?	Yes
**Resources**<br><br>A description of all resources used, including antibodies, cell lines, animals and software tools, with enough information to allow them to be uniquely	Yes

Powered by Editorial Manager® and ProduXion Manager® from Aries Systems Corporation

identified, should be included in the Methods section. Authors are strongly encouraged to cite Research Resource Identifiers (RRIDs) for antibodies, model organisms and tools, where possible.<br><br>Have you included the information requested as detailed in our Minimum Standards Reporting Checklist?	
**Availability of data and materials**<br><br>All datasets and code on which the conclusions of the paper rely must be either included in your submission or deposited in publicly available repositories (where available and ethically appropriate), referencing such data using a unique identifier in the references and in the "Availability of Data and Materials" section of your manuscript.<br><br>Have you have met the above requirement as detailed in our Minimum Standards Reporting Checklist?	Yes

Powered by Editorial Manager® and ProduXion Manager® from Aries Systems Corporation

Revised main manuscript

Click here to view linked References

Click here to access/download;Manuscript;bionitio_manuscript_revised.docx

Bionitio: demonstrating and facilitating best practices for bioinformatics command-line software

Peter Georgeson: Melbourne Bioinformatics, The University of Melbourne, Melbourne, Victoria, Australia. Department of Clinical Pathology, The University of Melbourne, Australia, peter.georgeson@unimelb.edu.au

Anna Syme: Melbourne Bioinformatics, The University of Melbourne, Melbourne, Victoria, Australia. Royal Botanic Gardens Melbourne, Victoria, Australia. anna.syme@rbg.vic.gov.au

Clare Sloggett: Melbourne Bioinformatics, The University of Melbourne, Melbourne, Victoria, Australia. sloc@unimelb.edu.au

Jessica Chung: Melbourne Bioinformatics, The University of Melbourne, Melbourne, Victoria, Australia. jchung@unimelb.edu.au

Harriet Dashnow: Bioinformatics, Murdoch Children's Research Institute, Royal Children's Hospital, Parkville, Victoria, Australia and School of BioSciences, The University of Melbourne, Melbourne, Victoria, Australia. harriet.dashnow@mcri.edu.au

Michael Milton: Melbourne Bioinformatics, The University of Melbourne, Melbourne, Victoria, Australia, Melbourne Genomics Health Alliance, Walter and Eliza Hall Institute, Parkville, Victoria, Australia. michael.milton@unimelb.edu.au

Andrew Lonsdale: ARC Centre of Excellence in Plant Cell Walls, School of BioSciences, The University of Melbourne, Parkville, Victoria, Australia and Bioinformatics, Murdoch Children's Research Institute, Royal Children's Hospital, Parkville, Victoria, Australia. andrew.lonsdale@lonsbio.com.au

David Powell: Monash Bioinformatics Platform, Biomedicine Discovery Institute, Faculty of Medicine, Nursing and Health Sciences, Monash University, Clayton, Victoria, Australia. david.powell@monash.edu

Torsten Seemann: Melbourne Bioinformatics, The University of Melbourne, Melbourne, Victoria, Australia; Department of Microbiology and Immunology, Doherty Institute for Infection and Immunity, The University of Melbourne, Melbourne, Victoria, Australia. t.seemann@unimelb.edu.au

Bernard Pope: Melbourne Bioinformatics, The University of Melbourne, Melbourne, Victoria, Australia. Department of Clinical Pathology, The University of Melbourne, Australia. Department of Medicine, Central Clinical School, Monash University, Australia. bjpope@unimelb.edu.au. (* Corresponding author)

// Abstract

Background

Bioinformatics software tools are often created *ad hoc*, frequently by people without extensive training in software development. In particular, for beginners, the barrier to entry in bioinformatics software development is high, especially if they want to adopt good programming practices. Even experienced developers do not always follow best practices in all the code they develop. A consequence of this is the proliferation of poorer-quality bioinformatics software, leading to limited scalability and inefficient use of resources; lack of reproducibility, usability, adaptability and interoperability; and erroneous or inaccurate results.

Findings

In response to this problem we have developed Bionitio, a tool that automates the process of starting new bioinformatics software projects following recommended best-practices. With a single command, the user can create a new well-structured project in one of twelve programming languages. The resulting software is functional, carrying out a prototypical bioinformatics task, and thus serves as both a working example and a template for building new tools. Key features include command line argument parsing, error handling, progress logging, defined exit status values, a test suite, a version number, standardised building and packaging, user documentation, code documentation, a standard open-source software license, software revision control, and containerisation.

Conclusions

Bionitio serves as a learning aid for beginner-to-intermediate bioinformatics programmers and provides an excellent starting point for new projects. This helps developers adopt good

programming practices from the beginning of a project and encourages high-quality tools to be developed more rapidly. This also benefits users of the tools because they are more easily installed and consistent in their usage. Bionitio is released as open source software under the MIT License, and is available at https://github.com/bionitio-team/bionitio.

Keywords

bioinformatics, software development, best practices, training

Findings

Background

Software development is a central part of Bioinformatics, spanning a wide gamut of activities including data transformation, scripting, workflows, statistical analysis, data visualisation, and the implementation of core analytical algorithms. However, despite the critical and far-reaching nature of this work, there is a high degree of variability in the quality of bioinformatics software tools being developed, reflecting a broader trend across all scientific disciplines [1–3].

A common approach to defining software quality is to consider how well it meets its requirements. These can be *functional* - identifying what the software should do, and *non-functional* - identifying how it should work. Given the results-driven nature of research, the functional aspects of scientific programs (e.g. whether expected inputs produce expected outputs) are heavily emphasised at the expense of the non-functional ones (e.g. usability, maintainability, interoperability, efficiency) [4]. Additionally, the highly complex and evolving nature of scientific software can make software requirements specifications (SRSs) infeasible, and therefore they are rarely defined in practice [4,5].

The underlying causes of poor bioinformatics software quality are multifaceted, however two important factors have been highlighted in the literature: 1) the lack of software engineering training amongst bioinformaticians [2,3,6–11]; and 2) the fact that research groups have limited time and money to spend on software quality assurance [10,12–15] . As a result many bad practices are recurrently observed in the field. Lack of code documentation and user support makes tools hard to install, understand and use. Limited or non-existent testing can result in unreliable and buggy behaviour. A high-degree of coupling with the local computing environment and software dependencies impedes portability. The consequences of poor quality software can have a significant impact on scientific outcomes. Substantial amounts of users' time can be wasted in trying to get programs to work, scientific methods can be difficult to reproduce, and in the worst-case, scientific results can be invalid due to program errors or incorrect usage [3,7,8,10,12,13,16,17] .

The above-mentioned problems are well known and have prompted remedial action in a number of areas. Activities to increase software development training amongst scientists are under way, the most notable examples being the highly successful Software Carpentry and Data Carpentry workshops [2,3] . Additionally, there are many useful recommendations in the literature offering practical advice for beginners [9,12,16,18] including specific advice for biologists new to programming [19]. Significant efforts have also been made in producing software package collections where best-practice guidelines and curation provide minimum standards of software quality, such as Bioconductor for R [20], and Bioconda for bioinformatics command-line tools [21], to name two prominent examples. Operating system virtualisation services, such as Docker [22], and workflow specification languages, such as the Common Workflow Language (CWL) [23], have improved portability and reproducibility of tools and pipelines [12,24–26]. Increasing the resources available for scientific software development remains a complex challenge. The Software Sustainability Institute in the UK demonstrates one successful model where pooled research funding enables the provision of

consultancy, training and advocacy for scientific software development on a national level [27].

In this work we adopt a pragmatic approach to improving bioinformatics software quality that is summarised by Rule 7 in Carey and Papin's *Ten simple rules for biologists learning to program*: "develop good habits early on" [19]. The idea is that new bioinformatics tools should be started by copying and modifying a well-written existing example. This allows bioinformaticians to get started quickly on solving the crux of their problem, but also ensures that all the ingredients of good programming style and functionality are present from the beginning. Based on this concept we have developed a tool called Bionitio that automates the process of starting new bioinformatics software projects with recommended software best-practices built-in. With a single command the user can create a new well-structured project in one of (currently) twelve programming languages. The resulting software is functional, carrying out a prototypical bioinformatics task, and thus serves as both a working example and a template for building new tools. It is expected that users will incrementally modify this program to ultimately satisfy the requirements of their task at hand. The key point is that they are building on solid foundations, and because a lot of the mundane-but-important boilerplate is provided by Bionitio, there are fewer barriers to adopting good practices from the start. Specifically, every new Bionitio-created project includes command line argument parsing, error handling, progress logging, defined exit status values, a test suite, a version number, standardised building and packaging, user documentation, code documentation, a standard open-source software license, software revision control, containerisation with Docker, and a CWL wrapper. In this paper we describe the design and implementation of Bionitio and demonstrate how it can be used to quickly start new bioinformatics projects.

The closest related work to Bionitio is the Cookiecutter project [28]. It also takes advantage of the templating approach for starting new software projects, but it is targeted at a different

audience. Cookiecutter provides a more general-purpose templating system that is best suited to starting new software systems in *specific* programming languages, such as the instantiation of web applications based on particular web framework libraries. Conversely, Bionitio provides many instances of the same prototypical bioinformatics tool implemented in *different* programming languages. While Bionitio could theoretically be implemented on top of a system such as Cookiecutter, we believe that the extra complexity is not warranted and would be a barrier to understanding for our target audience.

Design and Implementation

Bionitio is designed around two components.

The first component is a prototypical bioinformatics tool that has been re-implemented in (currently) twelve different programming languages. Each implementation of the tool carries out exactly the same task, and each is stored in its own repository on GitHub underneath the bionitio-team project. For example, the Python 3 and C++ implementations are found at the following GitHub URLs respectively:

https://github.com/bionitio-team/bionitio-python

https://github.com/bionitio-team/bionitio-cpp

Each of the repositories acts as a self-contained exemplar of how to implement the prototypical tool in the given programming language, carrying out good programming practices (e.g. command-line argument parsing) in a language-idiomatic way.

The second component is a "bootstrap" script that automates the process of creating a new software project based on one of the language-specific repositories. With a single invocation of the bootstrap script the user can quickly start a new project; all they need to do is specify

a new project name and the programming language to use (the $ sign indicates the command line prompt):

```
$ bionitio-boot.sh -n newproj -i python
```

The example above creates a new local repository called "newproj" on the user's computer by cloning and then renaming the bionitio-python repository. Optionally, the user can also specify their GitHub username, which will cause the bootstrap script to create and populate a remote repository on GitHub for the new project. The repository comes with a test-suite, allowing continuous integration testing to easily be enabled via GitHub's integration with Travis CI [29]. The overall process carried out by the bootstrap script is illustrated in Figure 1.

Figure 1. Overview of the automated process for creating new projects performed by the Bionitio bootstrap script.

The prototypical bioinformatics tool is intended to be easy to understand and modify. Therefore it has only minimal functionality; just enough to demonstrate all the key features of a real bioinformatics command line program without becoming distracted by unnecessary complexity. In essence, the tool streams input from one or more FASTA files, computes several simple statistics about each file, and prints a tabulated summary of results on standard output. For example, the command below illustrates the behaviour of the tool on a single input FASTA file called "file1.fa":

```
$ bionitio file1.fa
FILENAME  NUMSEQ  TOTAL    MIN  AVG  MAX
file1.fa  5264    3801855  31   722  53540
```

The output is in tab-delimited format, consisting of a header row, followed by one or more rows of data, one for each input file. Each data row contains the name of the input file, followed by the total number of sequences in the file (NUMSEQ), the sum of the length of all the sequences in the file (TOTAL), followed by the minimum (MIN), average (AVG), and maximum (MAX) sequence lengths encountered in the file. An optional command line argument --minlen can be supplied, causing the program to ignore sequences with length strictly less than the given value.

Each implementation is self-contained and ready to be installed and executed. Consequently, Bionitio is an excellent resource for programmer training. However, the main intended use-case is that Bionitio will be used as the starting point for new projects and we expect users to rewrite parts of it to carry out their own desired functionality. Given that much of the boilerplate is already provided, modifying the program should be significantly easier than starting from scratch.

The twelve current implementation languages were chosen to represent the most commonly used languages in bioinformatics [17] (C, C++, Java, Javascript, Perl, Python, R and Ruby) but also to provide examples in up-and-coming languages and paradigms (C#, Clojure, Haskell and Rust). The fact that each instance implements the same prototypical tool provides important consistency amongst the different instances. It means that they all have common functionality, they can be easily compared, they can share the same test suite, their user documentation in the form of a README file can be templated, and the inclusion of new programming language implementations is straightforward. Over time we hope that new language implementations will be contributed by the community.

All the components of Bionitio are released under the terms of the MIT license, however we explicitly grant users permission to choose their own license for derivative works. The bootstrap script optionally allows the user to choose one of several standard open source

licences for newly created projects (Apache-2.0, BSD-2-Clause, BSD-3-Clause, GPL-2.0, GPL-3.0 and MIT). If no license is specified the MIT is chosen as the default. The terms of the license are copied into the LICENSE file in the top level of the repository, and all references to the license in source files are updated accordingly.

The bootstrap script also accepts optional author name and email address arguments which, if supplied, are inserted into the source code and documentation files at appropriate places. Newly created projects are committed to fresh Git [30] repositories. All instances of the word "bionitio" are replaced with the new project name, including in file paths and file contents, and all files are checked into a new git repository with a pristine commit history.

In the remainder of this section we outline the main features incorporated into Bionitio's prototypical tool that facilitate good programming practices, and, where possible, relate them to the relevant recommendations in the literature. In the following section we demonstrate by example how Bionitio can be used to create a new software project.

Table 1 to appear here [See Additional file 1].

Command line argument parsing

We provide a standard command line interface that follows modern Unix conventions [31,32], including providing arguments for help (--help) and the program version (--version) [18,33], and provision of single-dash notation for short argument names and double-dash notation for long argument names. Most importantly, the help argument causes the program to display usage information, including a description of each argument and its expected parameters. Where possible we use standard library code for implementing command-line argument parsing (Table 1), which tends to simplify the process of adding new arguments and ensures that user help documentation is generated.

Input and output conventions and progress logging

Bioinformatics tools are often strung together in pipelines. A common Unix paradigm is that each tool should "expect the output of every program to become the input to another, as yet unknown, program" [34]. Consequently, the tool can take input from one or more files or from the standard input device (stdin), which may be piped from the output of another program. Similarly, output is written to the standard output device (stdout) in a tab-delimited format. Additionally, we ensure that error messages are always written to the standard error device (stderr) [18]).

We provide an optional progress logging facility (--log), providing useful metadata about a computation that can aid debugging and provenance [11]. Progress logging messages are written to a specified output file. The log file includes the command line used to execute the program, and entries indicating which files have been processed so far. Events in the log file are annotated with their date and time of occurrence. Where possible we use standard library code for the provision of logging services (Table 1), as these easily facilitate advanced features such as timestamping of log messages, log file roll-over, support for concurrency, and different levels of logging output (e.g. messages, warnings, errors, *etcetera*).

Library code for parsing common bioinformatics file formats

There are several tasks in bioinformatics that are common across analyses. For example, many tools will need to parse sequence files in FASTA format. Rather than re-write code for this, it is better to use existing libraries. "Don’t Repeat Yourself" is a maxim that can be applied at multiple levels when programming [11,12,35]. Millions of lines of high-quality open source software are freely available on the web. It is typically better to find an established

library or package that solves a problem than to attempt to write one's own routines for well-established problems [3] and this also improves reusability [10]. We demonstrate this principle by using existing bioinformatics library code to parse the input files (Table 1). This also allows Bionitio to demonstrate how non-standard library dependencies can be specified in the software package description, such as the "setup.py" file for Python that specifies a dependency on the biopython [36] library.

Defined exit status values

Processes on most operating systems return an integer exit status code upon termination. The Unix convention is to use zero for success and non-zero for error. Exit status values provide essential information about the behaviour of executed programs and are relied upon when programs are called within larger systems, such as bioinformatics pipelines. Such pipelines can become large and complex and can run for long periods of time, therefore the likelihood of errors is high. Improper indication of success or failure can have significant consequences for such systems. For example, erroneous reporting of exit status zero, for a computation that actually failed, can cause a pipeline to continue processing on incomplete results, yielding unpredictable behaviour, or worse, silent errors. Non-zero exit status values can also provide useful debugging information by distinguishing different classes of errors. Bionitio demonstrates good programming style by defining the exit status values as constants, and provides well-defined exit points in the program, and documents the meaning of the status values in the README file.

A test suite including unit tests, integration tests, and continuous integration

Software testing enables us to verify that the various components of the program work as expected, it allows us to modify the codebase while maintaining established functionality,

and provides examples that demonstrate how to use the software along with its expected behaviour [16].

Bionitio includes examples of both unit tests and integration tests. A unit test runs a single method in isolation and enables the verification that each method in the implementation behaves as expected without concern for its extended environment. Where possible we use unit testing library frameworks appropriate for each programming language because they offer significant extended functionality over hand-written tests, and can facilitate better output reporting (Table 1). Integration tests ensure that the program behaves correctly as an entire entity, with all the components working together. Given that all implementations of Bionitio are expected to behave in the same way, they all share the same underlying testing data and automated integration-testing shell script. The README file for the project shows how the user can run a simple test to ensure that the program is working as expected, which increases their confidence that it was installed correctly [12].

Continuous integration is a software development practice that requires all changes to a software project’s code base to be integrated, compiled and tested as changes are made. Travis is an online provider of continuous integration testing that enables automatic execution of tests whenever changes are committed to a source repository, and is currently available free to all GitHub users. This benefits software development by enabling any introduced problems to be identified faster [37], and avoids the introduction of breaking changes into the code. Each Bionitio implementation includes all the necessary Travis configuration files and demonstrates how continuous integration can be used to run both the unit and integration tests at each commit to the GitHub repository. The Bionitio wiki on GitHub contains detailed instructions about how to enable Travis for newly created projects. The README file also includes the URL to show the status badge for Travis testing, providing a quick way for users to see the status of continuous integration testing (for example, a green icon badge showing successful "build passing").

Version number

Version numbers allow users to track the provenance of their work [11,12,18]. This is particularly important in Science where reproducibility is a primary concern. Bionitio comes with a clearly defined version number which is defined as a constant in a single place in the source code, which can be displayed to the user of the program via the --version command line argument. We do not prescribe a particular versioning scheme to use (e.g. Semantic Versioning [38]), rather we prefer to let the user decide on the most appropriate mechanism for their work. Our main objective is that a version number is defined, that it can be easily discovered by the user, and that it is easy to update and modify in a single place in the program source code.

Standardised software packaging and containerisation

The installation process can be one of the most cumbersome and frustrating parts of using bioinformatics software, because many tools do not provide much assistance to the user [10], and complex dependency chains can clash with local settings [25]. Difficult to install software reduces reproducibility, is less likely to be used, and can cause problems with reliability due to differences between the developer and user computing environments. These problems can be addressed by using standard build tools and software packaging systems [12]. Such systems can automate the process of ensuring that correct and complete versions of software dependencies are installed [18], and by following conventional practice, they allow tools to integrate with the broader software ecosystem and follow the principle of least surprise [39]. Bionitio does this by adopting the idiomatic package and installation mechanisms for each implementation language. For example in Python we use Pip, in C we

use GNU autotools and make, and in C++ we use CMake. A full list of the building and packaging systems used in each implementation is provided in Table 1.

Standard packaging also helps with containerisation, which is becoming increasingly useful in bioinformatics [40]. Docker containers are a popular implementation of this concept, where the underlying operating system is virtualised and packaged alongside tools and their dependencies. This makes it easy to install "containerised" software on any platform that supports Docker, and facilitates reproducibility by enabling the exact same software build to be used on every system. Each Bionitio implementation comes with a "Dockerfile" that encodes all the necessary information needed to create a containerised version of the tool. As an added benefit, the Docker container is used in Travis Continuous Integration testing, which both simplifies the use of Travis and also enables the functionality of the container itself to be included in the tests.

A standard open-source software license

When software is distributed without a license it is generally interpreted to mean that no permission has been granted from the creators of the software to use, modify, or share it. This is counterproductive to adoption. A standard open-source license provides minimum fuss for users and increases the chances that software will be widely used [11], partly because it removes barriers to widespread access, and partly because it encourages transparency, reuse and collaboration [16]. It is very common for research centres to install software on behalf of their users. Unsurprisingly such research centres (and their parent institutions) tend to be risk averse when it comes to legal matters. A non-standard license is very likely to require vetting by lawyers, which can be a protracted exercise. Many license options are available [41]. As mentioned above, new projects started with Bionitio use the MIT license by default, but the user can choose from a number of standard options. The terms of the license are copied into the LICENSE file in the top level of the repository, and

the name of the license is indicated prominently in the README file, and in source code files.

Documentation

Software documentation broadly falls into two categories: user documentation that explains how to install and use the code, and developer documentation that explains how the program is designed and intended to work. For the intended use case of Bionitio we believe it is important to strike a balance between the extensiveness of documentation and the effort required to maintain it. Therefore we adopt pragmatic recommendations from the literature that offer a good compromise between cost and functionality.

For user documentation we provide two critical components: a README file that appears at the top level of the repository, and comprehensive command line usage output when via the --help argument [18,33,37] as discussed above. The README file includes a program description, dependencies, installation instructions, inputs and outputs, example usage, and licensing information [12,42]. To ease the burden of adding new implementations of Bionitio, and to ensure consistency across current implementations, we build each README file from a template, such that common parts of the documentation are shared, and language-specific details (such as installation instructions) can be instantiated as needed.

Good developer documentation tries to explain the reasoning behind the code rather than recapitulating its operations in text [3], and can improve code readability, usability and debugging [33]. In Bionitio we adopt the following conventions in each implementation. Every source code file begins with header documentation that contains at least the following information: the name of the module, a brief description of its purpose, copyright information (author names and date of creation), license information, and a maintainer email address, a concise summary of the main components and processes undertaken in the module. Author

names, creation dates, license name and maintainer email address can be automatically populated by the bootstrap script. Every non-trivial component of code (such as type definitions and procedures) are accompanied by a brief description of the purpose of the component, plus descriptions of the arguments and results of methods, including any conditions that are assumed to uphold.

Revision control

Software revision control provides a systematic way to manage software updates, allowing multiple branches of development to be maintained in parallel, and provides a critical means of coordinating groups of developers [11,12,37]. Modern revision control systems such as Git [30] provide flexible and scalable modes of collaboration, supporting individual programmers all the way up to large —- and potentially geographically distributed —- teams. The collaborative advantages of Git are complemented by the GitHub code hosting web application [43], currently the most popular repository for bioinformatics code [17]. GitHub adds issue tracking, documentation publishing, lightweight release management, integration with external tools such as continuous integration testing, and perhaps most importantly, an easy-to-use web interface for source browsing and discovery. Bionitio takes advantage of Git and GitHub in two ways. Firstly, the Bionitio project itself is hosted on GitHub, including each of the twelve language-specific implementations of our prototypical bioinformatics tool. The bootstrap script creates new projects by cloning from GitHub, and therefore GitHub acts as our web-accessible content management system. Where possible, common features amongst the implementations, such as testing data, are shared via Git submodules, avoiding repetition. Secondly, the bootstrap script makes it easy for users to create new GitHub-hosted projects by optionally automating the initialisation and population of new repositories via the GitHub API. This saves the user’s time, encourages the use of revision control from the start of the project, and facilitates sharing the code with collaborators.

Recommended programming conventions

Each implementation of Bionitio aims to follow the programming conventions of the implementation language. This includes the adoption of standard tools and libraries as well as adhering to programming style guidelines, such as PEP 8 in Python. By following these practices we enhance integration with the language ecosystem, avoid common pitfalls, and encourage contributions from external developers [37,44]. Where possible, we have adopted automated code formatting tools to ensure that we adhere to recommended style, and static analysis tools to identify likely infelicities and possible sources of error. A full list of the code formatting and static analysis tools used in each implementation is provided in Table 1.

CWL tool wrapper

Bioinformatics pipelines — where multiple tools are chained together to perform an overall analysis — create further challenges for reproducible science. This has motivated the creation of pipeline frameworks that allow the logic of such computations to be abstracted from the details of how they are executed. An emerging standard in this area is the Common Workflow Language (CWL) that is supported by several popular workflow engines. CWL comprises two declarative sub-languages: workflow descriptions, that define data flow patterns between pipeline stages; and command line tool descriptions, that define the interfaces of tools in a platform independent manner. Each Bionitio implementation provides a CWL tool description "bionitio.cwl", that facilitates its incorporation into CWL pipelines, and takes advantage of CWL's support for invoking programs within Docker containers.

Methods

In this section we demonstrate how to create a new bioinformatics software project using the Bionitio bootstrap script. In order to follow this process the user requires a GitHub account, and installation of Git on their local computer.

Step 1: choose a programming language, project name, and software license

The Bionitio prototypical bioinformatics tool is currently implemented in twelve programming languages: C, C++, C#, Clojure, Java, Javascript, Haskell, Perl5, Python, R, Ruby, or Rust. The user must choose which of these languages they want to use for their new project. For users relatively new to programming, with no prior constraints on their choice of language, we recommend they choose a high level interpreted language such as Python or R. The user must also choose a new name for their project. Optionally, the user may also choose an open source license for their code. The current available options are Apache-2.0, BSD-2-Clause, BSD-3-Clause, GPL-2.0, GPL-3.0 and MIT. If no license is specified, the MIT license is selected by default. In this example we will assume that Python is chosen as the implementation language, the project name is "newproj", and the BSD-3-Clause license is desired.

Step 2: run the bootstrap script to create a new software repository

The Bionitio bootstrap script is a BASH shell script that automates the process of creating new projects. In principle, if Bionitio is already installed on the user's computer, then the bootstrap script can be run like so:

```
$ bionitio-boot.sh -i python -n newproj -c BSD-3-Clause
```

A user may find it inconvenient to have Bionitio installed just to run the bootstrap script, therefore they may instead prefer to use Curl [45] to simplify the process, by downloading the script directly from GitHub before running it locally:

```
$ URL=https://raw.githubusercontent.com/\
bionitio-team/bionitio/master/boot/bionitio-boot.sh
$ curl -sSf $URL | bash -s -- -i python -n newproj -c BSD-3-Clause
```

Alternatively, the bootstrap script can be run from a Docker container published on DockerHub (https://cloud.docker.com/u/bionitio/repository/docker/bionitio/bionitio-boot):

```
$ docker run -it -v "$(pwd):/out" --rm bionitio/bionitio-boot \
-i python -n newproj -c BSD-3-Clause
```

The user may optionally specify an author name and email address, which will be substituted for placeholders in the source code and documentation at appropriate places:

```
$ bionitio-boot.sh -i python -n newproj -c BSD-3-Clause \
-a "Example Author" -e example.email@institute.org
```

Finally, the user may specify a GitHub username. In this circumstance the bootstrap script will create a new remote repository under the specified project name on GitHub and push the project to that repository:

```
$ bionitio-boot.sh -i python -n newproj -c BSD-3-Clause \
-a "Example Author" -e example.email@institute.org -g example_github_user
```

Step 3: run the test suite, and optionally setup continuous integration testing

Each new repository created by the bootstrap script contains a testing directory called "functional_tests". Within that directory is an automated testing shell script called (in this example) "newproj-test.sh" and a sub-directory of test data and corresponding expected outputs. The test script can be run like so:

```
$ newproj-test.sh -p newproj -d test_data
```

The test script reports how many tests passed and failed, and an optional -v (to enable verbose mode) will cause it to report more details about each test case that is run. Obviously, the test cases are specific to the expected behaviour of the prototypical bioinformatics tool implemented by Bionitio. It is expected that the user will replace these tests to suit the requirements of their new project. Despite this, the user will benefit from much of the testing infrastructure provided by the script.

If the user has created a remote repository for their project on GitHub, they can quickly enable continuous integration testing via Travis CI. Each new project created by Bionitio includes the necessary Travis configuration files that are needed to install the prototypical bioinformatics tool and run the integration and unit test scripts.

From this point onwards we expect that the user will go on to modify the program in order to carry out their intended task. This includes changing the code of the program itself, updating library dependencies, and importantly, adding appropriate test cases.

Conclusions

Software development is a complex task, involving many concepts and processes that can be daunting for beginners. Many bioinformaticians are not trained in software engineering, and research-oriented projects have limited budgets for quality assurance. The results-

driven focus of science means that many important non-functional software requirements are often overlooked. Unfortunately these factors mean that shortcuts are often taken for the sake of making something "that works", leading to a proliferation of lower-quality bioinformatics tools.

Bionitio takes a pragmatic approach to addressing this problem. Our ambition is to help beginner and intermediate bioinformaticians develop good habits early on. We aim to achieve this by automating much of the drudgery involved in setting up new projects by providing a simple working example that has the necessary boilerplate in place. By providing a fast and simple way to start new projects from solid foundations we believe that good practices are more likely to be adopted.

The challenges faced by the bioinformatics and science communities in building better quality software are well known, and there is no shortage of practical recommendations to be found in the literature. These guidelines are undoubtedly useful to beginners, however, we believe they fall short in two ways. First, they are spread over multiple manuscripts that only partially overlap in their recommendations, therefore some level of consolidation is needed. Second, they are static artefacts that point to good practices but do not remove the considerable burden of applying them in real code. These two observations motivated the creation of Bionitio, both as a way of collecting commonly recommended best practices, and as a way of demonstrating and facilitating their use. Therefore a significant contribution of our work is to build a tool that can both illustrate best practices by example but also make it easy to use them in new projects. In this sense Bionitio takes a much more active role in the dissemination and compliance with these principles.

Role in education and training

In very recent work Tractenberg *et al* have developed a Mastery Rubric for Bioinformatics with the goal of better defining skills development and competencies in the discipline [46]. In this framework, competency in computational methods ranges through five levels, from novice (stage 1) to independent bioinformatics practitioner (stage 5). One of the goals of Bionitio is to support education and training for advancing bioinformaticians from stage 3 - learning best practices in programming, and writing basic code - to stage 5 - developing new software that is useful, efficient, standardized, well-documented and reproducible. As an example of this application, Bionitio was used as the basis for a whole-day workshop on best practices in bioinformatics software development at the Australian Bioinformatics and Computational Biology Society (ABACBS) Annual Conference in November 2018 [47], delivered to an audience of 50 bioinformaticians from research and clinical institutes around Australia. In the first half of the workshop participants learnt how to set up a new software repository using Bionitio, allowing time for exploration of the codebase, discussion of key aspects of quality software, and an explanation of the processes that are automated by Bionitio. In the second half of the workshop participants learnt about test-driven development (TDD) and undertook an exercise to extend the codebase with new features, documentation, corresponding test cases, and linkage to revision control and continuous integration testing. In this setting, Bionitio's design as a simple-yet-realistic bioinformatics exemplar provides both a common codebase for coordination of workshop materials and an extensible platform for the delivery of hands-on practical activities. Additionally, by providing complete working examples in many different languages, Bionitio acts as a kind of "Rosetta Stone" and is therefore likely an excellent vehicle for comparative programming skills transfer.

Alignment with FAIR Principles and OSS Recommendations

In an effort to facilitate continued benefit from the digital assets related to data-intensive science, representatives from academia, industry, funding agencies, and publishers have proposed the FAIR Data Principles that aim to make experimental artefacts findable,

accessible, interoperable and reusable for machines and people [48]. Jiménez *et al* have argued that poor development practices result in lower quality outputs that negatively impact reproducibility and reusability of research [49], and propose four principles for open source software development (OSS recommendations) that align well with the FAIR principles: 1) make source code publicly accessible from day one; 2) make software easy to discover by providing software metadata via a popular community registry; 3) adopt a licence and comply with the licence of third-party dependencies; and 4) define clear and transparent contribution, governance and communication processes. Tools developed with Bionitio have a head start on satisfying both the FAIR principles and the first three OSS recommendations:

- they are publicly accessible in GitHub repositories with clearly indicated standard open source licences and user documentation;
- they are interoperable with other tools via standardised inputs and outputs and interfaces that follow long-established conventions;
- they are re-usable by virtue of the adoption of standard build procedures, the provision of clear documentation relating to installation and usage, containerisation with Docker, and integration into CWL;
- where appropriate, specific versions (with defined version numbers) can be made findable by the allocation of Digital Object Identifiers facilitated by Zenodo [50] through GitHub.

Importantly, Bionitio facilitates *compliance* with these principles, which is seen by Jiménez *et al* as the final (and, in our opinion, most difficult) step in organisational adoption.

Availability of supporting source code and requirements

- Project name: Bionitio
- Project home page: https://github.com/bionitio-team/bionitio
- Operating system(s): Any POSIX-like system.
- Programming language: Users can choose from: C, C++, C#, Clojure, Java, Javascript, Haskell, Perl, Python, R, Ruby, Rust
- Other requirements: BASH, curl and git are required for bionitio-boot.sh.
- License: MIT
- RRID: SCR_017259

Declarations

Ethics approval and consent to participate: Not applicable

Consent for publication: Not applicable

Competing interests: The authors declare that they have no competing interests.

Funding: BP is supported by a Victorian Health and Medical Research Fellowship. HD is supported by an Australian Government Research Training Program (RTP) Scholarship, an Australian Genomics Health Alliance top up scholarship and a Murdoch Children's Research Institute top up scholarship. AL is supported by an Australian Government Research Training Program (RTP) Scholarship. PG is supported by an Australian Government Research Training Program (RTP) Scholarship.

Authors' contributions: TS, AL, HD and BP conceived of the project. All authors contributed to the design, implementation, testing and documentation of Bionitio. AS, CS, AL, HD, PG and BP contributed to manuscript drafting. All authors contributed to manuscript proofreading and final editing.

Acknowledgements: The authors would like to thank Melbourne Bioinformatics for providing computing resources for the development of Bionitio, and to the many users of the tool who have provided feedback about its use.

References

1. Baker M. 1,500 scientists lift the lid on reproducibility. Nature. 2016;533:452–4.

2. Wilson G. Software Carpentry: lessons learned. F1000Res. 2014;3:62.

3. Wilson G, Aruliah DA, Brown CT, Chue Hong NP, Davis M, Guy RT, et al. Best practices for scientific computing. PLoS Biol. 2014;12:e1001745.

4. Verma D, Gesell J, Siy H, Zand M. Lack of software engineering practices in the development of bioinformatics software. ICCGI. 2013;2013:57–62.

5. Segal J, Morris C. Developing Scientific Software. IEEE Softw. 2008;25:18–20.

6. Hannay JE, MacLeod C, Singer J, Langtangen HP, Pfahl D, Wilson G. How Do Scientists Develop and Use Scientific Software? Proceedings of the 2009 ICSE Workshop on Software Engineering for Computational Science and Engineering. Washington, DC, USA: IEEE Computer Society; 2009. p. 1–8.

7. Merali Z. Error: why scientific programming does not compute. Nature. 2010;467:775–7.

8. Joppa LN, McInerny G, Harper R, Salido L, Takeda K, O'Hara K, et al. Troubling Trends in Scientific Software Use. Science. American Association for the Advancement of Science; 2013;340:814–5.

9. Baxter SM, Day SW, Fetrow JS, Reisinger SJ. Scientific software development is not an oxymoron. PLoS Comput Biol. 2006;2:e87.

10. Lawlor B, Walsh P. Engineering bioinformatics: building reliability, performance and productivity into bioinformatics software. Bioengineered. 2015;6:193–203.

11. List M, Ebert P, Albrecht F. Ten Simple Rules for Developing Usable Software in Computational Biology. PLoS Comput Biol. 2017;13:e1005265.

12. Taschuk M, Wilson G. Ten simple rules for making research software more robust. PLoS Comput Biol. 2017;13:e1005412.

13. Prins P, de Ligt J, Tarasov A, Jansen RC, Cuppen E, Bourne PE. Toward effective software solutions for big biology. Nat Biotechnol. 2015;33:686–7.

14. Umarji M, Seaman C, Gunes Koru A, Liu H. Software Engineering Education for Bioinformatics [Internet]. 2009 22nd Conference on Software Engineering Education and Training. 2009. Available from: http://dx.doi.org/10.1109/cseet.2009.44

15. Howison J, Deelman E, McLennan MJ, Ferreira da Silva R, Herbsleb JD. Understanding the scientific software ecosystem and its impact: Current and future measures. Res Eval. Narnia; 2015;24:454–70.

16. Leprevost F da V, Barbosa VC, Francisco EL, Perez-Riverol Y, Carvalho PC. On best practices in the development of bioinformatics software. Front Genet. 2014;5:199.

17. Russell PH, Johnson RL, Ananthan S, Harnke B, Carlson NE. A large-scale analysis of bioinformatics code on GitHub. PLoS One. 2018;13:e0205898.

18. Seemann T. Ten recommendations for creating usable bioinformatics command line software. Gigascience. 2013;2:15.

19. Carey MA, Papin JA. Ten simple rules for biologists learning to program. PLoS Comput Biol. 2018;14:e1005871.

20. Huber W, Carey VJ, Gentleman R, Anders S, Carlson M, Carvalho BS, et al. Orchestrating high-throughput genomic analysis with Bioconductor. Nat Methods. 2015;12:115–21.

21. Grüning B, Dale R, Sjödin A, Chapman BA, Rowe J, Tomkins-Tinch CH, et al. Bioconda: sustainable and comprehensive software distribution for the life sciences. Nat Methods. 2018;15:475–6.

22. Docker [Internet]. [cited 2019 Jul 8]. Available from: https://www.docker.com/

23. Amstutz P, Crusoe MR, Tijanić N, Chapman B, Chilton J, Heuer M, et al. Common Workflow Language, v1.0 [Internet]. 2016. Available from: https://doi.org/10.6084/m9.figshare.3115156.v2

24. Jackman SD, Mozgacheva T, Chen S, O'Huiginn B, Bailey L, Birol I, et al. ORCA: A Comprehensive Bioinformatics Container Environment for Education and Research. Bioinformatics [Internet]. 2019; Available from: http://dx.doi.org/10.1093/bioinformatics/btz278

25. Belmann P, Dröge J, Bremges A, McHardy AC, Sczyrba A, Barton MD. Bioboxes: standardised containers for interchangeable bioinformatics software [Internet]. GigaScience. 2015. Available from: http://dx.doi.org/10.1186/s13742-015-0087-0

26. O'Connor BD, Yuen D, Chung V, Duncan AG, Liu XK, Patricia J, et al. The Dockstore: enabling modular, community-focused sharing of Docker-based genomics tools and workflows. F1000Res. 2017;6:52.

27. Crouch S, Hong NC, Hettrick S, Jackson M, Pawlik A, Sufi S, et al. The Software Sustainability Institute: Changing Research Software Attitudes and Practices. Comput Sci Eng. IEEE Computer Society; 2013;15:74–80.

28. Greenfeld AR. Cookiecutter [Internet]. Available from: https://github.com/audreyr/cookiecutter

29. Travis CI - Test and Deploy Your Code with Confidence [Internet]. [cited 2019 Mar 21]. Available from: https://travis-ci.org/

30. The Git Project. Git [Internet]. Git. [cited 2019 Apr 14]. Available from: https://git-scm.com/

31. IEEE and The Open Group. The Open Group Base Specifications Issue 7, 2018 edition [Internet]. 2018. Report No.: 1003.1-2008. Available from: http://pubs.opengroup.org/onlinepubs/9699919799/basedefs/V1_chap12.html

32. Free Software Foundation, Inc. GNU Coding Standards [Internet]. 2019 Feb. Available from: https://www.gnu.org/prep/standards/standards.html

33. Lee BD. Ten simple rules for documenting scientific software. PLoS Comput Biol. 2018;14:e1006561.

34. McIlroy MD, Pinson EN, Tague BA. UNIX Time-Sharing System: Foreword. Bell System

Technical Journal. Wiley Online Library; 1978;57:1899–904.

35. Andrew H, David T. The Pragmatic Programmer: From Journeyman to Master. Addison Wesley Longman, Redwood City; 2000.

36. Cock PJA, Antao T, Chang JT, Chapman BA, Cox CJ, Dalke A, et al. Biopython: freely available Python tools for computational molecular biology and bioinformatics. Bioinformatics. 2009;25:1422–3.

37. Karimzadeh M, Hoffman MM. Top considerations for creating bioinformatics software documentation. Brief Bioinform. 2018;19:693–9.

38. Preston-Werner T. Semantic Versioning 2.0.0 [Internet]. [cited 2019 Mar 4]. Available from: https://semver.org/spec/v2.0.0.html

39. Raymond ES. The Art of UNIX Programming. Addison-Wesley Professional; 2003.

40. Gruening B, Sallou O, Moreno P, da Veiga Leprevost F, Ménager H, Søndergaard D, et al. Recommendations for the packaging and containerizing of bioinformatics software. F1000Res [Internet]. 2019 [cited 2019 Mar 31];7. Available from: https://f1000research.com/articles/7-742/v2/pdf

41. Choose an open source license [Internet]. [cited 2019 Mar 4]. Available from: https://choosealicense.com/

42. Johnson M. Building a Better ReadMe. Technical Communication. 1997;44:28–36.

43. Perez-Riverol Y, Gatto L, Wang R, Sachsenberg T, Uszkoreit J, Leprevost F da V, et al. Ten Simple Rules for Taking Advantage of Git and GitHub. PLoS Comput Biol. 2016;12:e1004947.

44. Glass RL. Facts and Fallacies of Software Engineering. Addison-Wesley Professional; 2003.

45. The Curl developers. Curl [Internet]. Curl: command line tool and library for transferring data with URLs. [cited 2019 Apr 14]. Available from: https://curl.haxx.se/

46. Tractenberg RE, Lindvall JM, Attwood TK, Via A. The Mastery Rubric for Bioinformatics: supporting design and evaluation of career-spanning education and training. bioRxiv [Internet]. biorxiv.org; 2019; Available from: https://www.biorxiv.org/content/10.1101/655456v1.abstract

47. ABACBS-2018 Annual Conference [Internet]. [cited 18 June, 2019]. Available from: https://www.abacbs.org/conference2018/about

48. Wilkinson MD, Dumontier M, Aalbersberg IJJ, Appleton G, Axton M, Baak A, et al. The FAIR Guiding Principles for scientific data management and stewardship. Sci Data. 2016;3:160018.

49. Jiménez RC, Kuzak M, Alhamdoosh M, Barker M, Batut B, Borg M, et al. Four simple recommendations to encourage best practices in research software. F1000Res [Internet]. 2017;6. Available from: http://dx.doi.org/10.12688/f1000research.11407.1

50. Zenodo [Internet]. [cited 18 June, 2019]. Available from: https://zenodo.org/

Additional Files

File name: Additional file 1

File format: Microsoft Word

Title of data: Table 1

Description of data: Contents of Table 1 to be included in the manuscript where indicated.

Table Click here to access/download;Table;Additional file 1.docx

language	build/deploy	FASTA reading	command line argument parsing	unit testing	logging	static analysis	code format
C	make	kseq	getopt	assert	custom	lint	clang-format
C++	cmake	Seqan	boost::program_options	catch	boost::log	cppcheck	clang-format
C#	dotnet	.Net Bio	Microsoft.Extensions.CommandLineUtils	Microsoft.VisualStudio.TestTools.UnitTesting	Serilog	N/A	N/A
Clojure	lieningen	Bioclojure	clojure.tools.cli	clojure.test	timbre	Eastwood	cljfmt
Java	maven	biojava	Apache Commons	junit	custom	checkstyle	checkstyle
Javascript	node	fasta-parser	commander	mocha	winston	N/A	standard
Haskell	stack	BioHaskell	optparse-applicative	hspec	hslogger	hlint	N/A
Perl	N/A	BioPerl	Getopt::ArgParese	Test::More	Log::Log4perl	perlcritic	perltidy
Python	pip	biopython	argparse	unittest	logging	pylint	N/A
R	R	seqinr	optparse	testthat	logging	lintr	N/A
Ruby	gem	bioruby	optparse	Test::Unit	logger	N/A	N/A
Rust	cargo	bio::io::fasta	argparse	native test feature of Rust	log, log4rs	N/A	rustfmt

Table 1. Standard libraries and tools employed by each implementation of Bionitio. Instances where no appropriate option was available are marked with N/A.

Figure

Click here to access/download;Figure;figure1.png

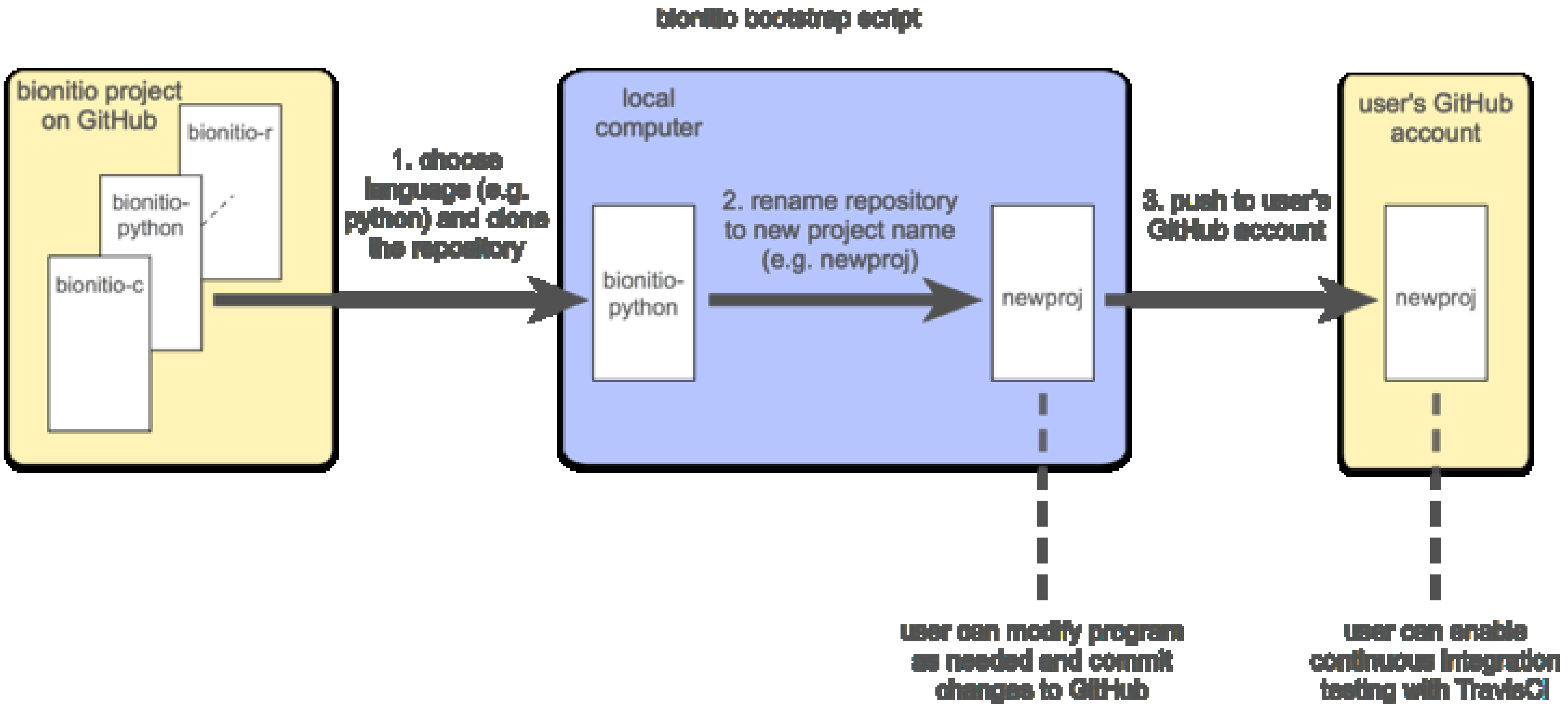

SVG version of figure1.png

Click here to access/download
Supplementary Material
figure1.svg

Response to reviewer comments

Click here to access/download;Personal Cover;bionitio_response_to_reviewers.pdf

16 July 2019

To the Editor-in-Chief and Executive Editor, GigaScience,

We thank the Editor and Reviewers for their insightful and constructive comments on our submission "Bionitio: demonstrating and facilitating best practices for bioinformatics command-line software" (GIGA-D-19-00145). In the following document we respond to each of the reviewer comments and say what has changed in the software and text to address each point. In the revised manuscript we have used red font to indicate changes that we have made to the body of the text. We believe that the suggested changes have significantly improved the quality of the paper and the corresponding Bionitio tool.

The responses here are presented in the order that the comments appear in the manuscript review.

Editor Comments

> The reviewers agree that the tool itself is a useful contribution, overall. However, they also have some constructive suggestions for improving the manuscript.
>
> In particular, I agree with reviewer 1 that, ideally, the manuscript should also present "an evidence-backed testimony about the tool's efficacy in correcting the problems stated in the introduction."
> I understand that a typical, quantitative benchmarking exercise may not be possible for this type of tool, but reviewer 1 has some good pointers regarding issues that should be discussed in more detail (for example, regarding FAIR principles and how your approach suggested in the paper can help in this regard).
>
> Reviewer 2 has some notes on installation and running the tool that may give you some hints for minor improvements or corrections.
>
> The reviewers also suggest to provide the tool via a container (e.g. docker), especially as it is meant to be helpful for beginners.
>
> In addition, please register any new software application in the SciCrunch.org database to receive a RRID (Research Resource Identification Initiative ID) number, and include this in your manuscript. This will facilitate tracking, reproducibility and re-use of your tool.

Response

We concur with the Editor that a qualitative benchmarking exercise is challenging for this type of tool, and that a detailed discussion of our alignment with FAIR principles is a valuable contribution to the paper. In light of these remarks we have included a section on how Bionitio enables bioinformaticians to easily adopt many of the key FAIR principles and have additionally linked this to recent work on related recommendations for Open Source research software. Disclaimer: a Bionitio author (B Pope) a co-author on the latter recommendations.

We have attempted to address issues related to installation and running the tool outlined by Reviewer 2 and have provided Docker containers for each of the Bionitio implementations, as well as the bootstrap script, and updated the documentation accordingly.

We have applied for registration of Bionitio through SciCrunch.org and received an RRID of SCR_017259. We have included this in the manuscript.

Reviewer 1

Comment 1 (and comment 12)

> The limitation of this manuscript, in my mind, is mostly that it reads like more of an instruction manual and list of general best practices than a detailed technical write up about the contribution made, and an evidence-backed testimony about its efficacy in correcting the problems stated in the introduction.

Response

We believe that one of the contributions of Bionitio is that it provides a consolidation of many disparate sources of best practices for software development in bioinformatics. Indeed, the features present in Bionitio are distilled from more than 25 different partially overlapping recommendations. We also believe that it is a contribution of our manuscript to explicitly link those recommendations to the features present in our tool. Therefore, there is necessity to list our sources and argue for their significance. Another key contribution of our paper is to show how easily a new project can be created with our tool, as a step-by-step guide to its main features.

However, we also agree that our manuscript could have made our contributions clearer and argued further for its efficacy in correcting the problems stated in the introduction.

We also agree with the Editor that "a typical, quantitative benchmarking exercise may not be possible for this type of tool".

In light of these comments we have made considerable changes to the manuscript.

The following text was added to the conclusion to show how Bionitio helps users to adopt FAIR principles and related recommendations for open source software:

> **Alignment with FAIR Principles and OSS Recommendations**
>
> In an effort to facilitate continued benefit from the digital assets related to data-intensive science, representatives from academia, industry, funding agencies, and publishers have proposed the FAIR Data Principles that aim to make experimental artefacts findable, accessible, interoperable and reusable for machines and people [48]. Jiménez *et al* have argued that poor development practices result in lower quality outputs that negatively impact reproducibility and reusability of research [49], and propose four principles for open source software development (OSS recommendations) that align well

with the FAIR principles: 1) make source code publicly accessible from day one; 2) make software easy to discover by providing software metadata via a popular community registry; 3) adopt a licence and comply with the licence of third-party dependencies; and 4) define clear and transparent contribution, governance and communication processes. Tools developed with Bionitio have a head start on satisfying both the FAIR principles and the first three OSS recommendations:

- they are publicly accessible in GitHub repositories with clearly indicated standard open source licences and user documentation;
- they are interoperable with other tools via standardised inputs and outputs and interfaces that follow long-established conventions;
- they are re-usable by virtue of the adoption of standard build procedures, the provision of clear documentation relating to installation and usage, containerisation with Docker, and integration into CWL;
- where appropriate, specific versions (with defined version numbers) can be made findable by the allocation of Digital Object Identifiers facilitated by Zenodo [50] through GitHub.

Importantly, Bionitio facilitates *compliance* with these principles, which is seen by Jiménez *et al* as the final (and, in our opinion, most difficult) step in organisational adoption.

The following text was added to the conclusion to outline Bionitio's role in education and training by relating it to the Mastery Rubric for Bioinformatics proposed by Tractenberg *et al*, along with our own experience in using it to deliver a national bioinformatics workshop (in Australia):

Role in education and training

In very recent work Tractenberg *et al* have developed a Mastery Rubric for Bioinformatics with the goal of better defining skills development and competencies in the discipline [46]. In this framework, competency in computational methods ranges through five levels, from novice (stage 1) to independent bioinformatics practitioner (stage 5). One of the goals of Bionitio is to support education and training for advancing bioinformaticians from stage 3 - learning best practices in programming and writing basic code - to stage 5 - developing new software that is useful, efficient, standardized, well-documented and reproducible. As an example of this application, Bionitio was used as the basis for a whole-day workshop on best practices in bioinformatics software development at the Australian Bioinformatics and Computational Biology Society (ABACBS) Annual Conference in November 2018 [47], delivered to an audience of 50 bioinformaticians from research and clinical institutes around Australia. In the first half of the workshop participants learnt how to set up a new software repository using Bionitio, allowing time for exploration of the codebase, discussion of key aspects of quality software, and an explanation of the processes that are automated by Bionitio. In the second half of the workshop participants learnt about test-driven development (TDD) and undertook an exercise to extend the codebase with new features, documentation, corresponding test cases, and linkage to revision control and continuous integration testing. In this setting, Bionitio's design as a simple-yet-realistic bioinformatics exemplar provides both a common codebase for coordination of workshop materials and an extensible platform for the delivery of hands-on practical activities. Additionally, by providing complete working examples in many different languages, Bionitio acts as a kind of "Rosetta Stone" and is therefore likely an excellent vehicle for comparative programming skills transfer.

We have also expanded the third paragraph in the Conclusions to emphasise why we think Bionitio is a significant contribution on top of the already existing recommendations in the literature (and the main motivation for its creation):

> The challenges faced by the bioinformatics and science communities in building better quality software are well known, and there is no shortage of practical recommendations to be found in the literature. These guidelines are undoubtedly useful to beginners, however we believe they fall short in two ways. First, they are spread over multiple manuscripts that only partially overlap in their recommendations, therefore some level of consolidation is needed. Second, they are static artefacts that point to good practices but do not remove the considerable burden of applying them in real code. These two observations motivated the creation of Bionitio, both as a way of collecting commonly recommended best practices, and as a way of demonstrating and facilitating their use. Therefore, a significant contribution of our work is to build a tool that can both illustrate best practices by example but also make it easy to use them in new projects. In this sense Bionitio takes a much more active role in the dissemination and compliance with these principles.

We have also emphasised the contribution that this tool makes to improving software development in bioinformatics as per comments 13,14,15 and 21 below.

Comment 2

> (section 1; paragraph 2) How is "correctness" evaluated in your mind? In research truth is often unknown by definition, so perhaps choose a less loaded word or elaborate on how this is evaluated.

Response

We agree with the reviewer that truth can be elusive in science, and therefore by correctness we mean that the software implements its intended functionality; so it is correct in the sense that it meets its specification (whether that specification be formally defined, or, more likely, part of the informal intentions that are known to the author(s)). Following the advice of the reviewer we have used a less loaded way to describe this, and changed the manuscript as follows:

> Given the results-driven nature of research, the functional aspects of scientific programs (e.g. ~~correctness~~ whether expected inputs produce expected outputs) are heavily emphasised at the expense of the non-functional ones (e.g. usability, maintainability, interoperability, efficiency).

Comment 3

> Duplicate heading at start of paper? Both "Findings" and "Background"

Response

> We believe that this formatting follows the suggested GigaScience Technical Note style (https://academic.oup.com/gigascience/pages/technical_note), where in the main text, "Findings" is a larger heading including the subheadings Background, Implementation, Methods, Conclusions, etc. If our interpretation of the formatting guidelines is incorrect, we are confident that this can be fixed in the final proof.

Comment 4

(section 1; paragraph 2, last sentence) Some "specifications" or recommendations, such as Nature Publishing's software checklist, and some 10-simple-rules articles in pnas related to scientific software. Are these the types of things you're referring to? If so, might be worth mentioning how they can exist but perhaps are harder to define for a specific (quickly moving) domain beyond the "basics".

Response

In this part we are referring to "software requirements specifications" that are commonly used in Software Engineering to define the functional and non-functional requirements of software being developed. We have changed the text to "software requirements specifications (SRSs)" to clarify this point.

Comment 5

(section 1; paragraph 4) abovementioned -> above-mentioned

Response

Corrected.

Comment 6

(section 1; second-last paragraph) "more likely to adopt good practices" <- have you witnessed this in the wild with bionitio, yet? I agree that in principle I'd expect this result, but giving students or researchers the tool and saying nothing else, then coming back at the end of the process, is this the outcome we get? The biggest places I see this not continuing beyond the boilerplate is documentation and testing. This could potentially also be answered if Cookiecutter has successes that you could reference.

Response

We agree with the reviewer that this is an expected result, however we have not formally tested it, and, for now it sits here as a hypothesis. We have reworded the sentence to make this point clearer:

The key point is that they are building on solid foundations, and because a lot of the mundane-but-important boilerplate is provided by Bionitio, there are fewer barriers to adopting good practices from the start.

Comment 7

(command line argument parsing) have you considered integrating these command-line descriptions with standard tools for shipping workflows to C(G)PUs, like Common Workflow Language (commonwl.org), Boutiques (boutiques.github.io), or others? It would be an additional feature you could add on top of each language-specific implementation that would make not only consuming the tools even more uniform, but enable scaling them out for large datasets more accessible for developers.

Response

We agree with the reviewer that this would be a useful additional feature, and therefore have added example CWL tool wrappers for each implementation of Bionitio. This addition was greatly facilitated by the fact that each Bionitio implementation has the same command line interface, and (now) comes with a Docker container. We have updated the online documentation for Bionitio to include information about this, and have made the following changes to the manuscript:

In the Background section:

> Operating system virtualisation services, such as Docker [22], and workflow specification languages, such as the Common Workflow Language (CWL) [23], have improved portability and reproducibility of tools and pipelines [12,24–26].
> ...
>
> Specifically, every new Bionitio-created project includes … containerisation with Docker, and a CWL wrapper.

In the Design and Implementation section:

> **CWL tool wrapper**
>
> Bioinformatics pipelines — where multiple tools are chained together to perform an overall analysis — create further challenges for reproducible science. This has motivated the creation of pipeline frameworks that allow the logic of such computations to be abstracted from the details of how they are executed. An emerging standard in this area is the Common Workflow Language (CWL) that is supported by several popular workflow engines. CWL comprises two declarative sub-languages: workflow descriptions, that define data flow patterns between pipeline stages; and command line tool descriptions, that define the interfaces of tools in a platform independent manner. Each Bionitio implementation provides a CWL tool description "bionitio.cwl", that facilitates its incorporation into CWL pipelines, and takes advantage of CWL's support for invoking programs within Docker containers.

We have also updated the README.md files for each implementation of Bionitio to include information about how to use the CWL tool wrapper and included running the CWL tool wrapper within Travis CI testing.

Comment 8

> (software packaging) there is also no mention of virtualization/containerization here, such as Docker or Singularity, that would also increase the portability of these packages. Have the authors considered this to further minimize this issue?

Response

We agree with the reviewer that this would be a useful additional feature, and therefore have added example Docker container definitions for each implementation of Bionitio, and also the bootstrap script. We have made the following changes to the manuscript:

In the Abstract:

Key features include … , and containerisation.

In the Background section:

Specifically, every new Bionitio-created project includes … containerisation with Docker, and a CWL wrapper.

In the Design and Implementation section:

Sub-heading changed from "Standardised software packaging using programming language specific mechanisms" to "Standardised software packaging and containerisation".

Text added:

Standard packaging also helps with containerisation, which is becoming increasingly useful in bioinformatics [40]. Docker containers are a popular implementation of this concept, where the underlying operating system is virtualised and packaged alongside tools and their dependencies. This makes it easy to install "containerised" software on any platform that supports Docker, and facilitates reproducibility by enabling the exact same software build to be used on every system. Each Bionitio implementation comes with a "Dockerfile" that encodes all the necessary information needed to create a containerised version of the tool. As an added benefit, the Docker container is used in Travis Continuous Integration testing, which both simplifies the use of Travis and also enables the functionality of the container itself to be included in the tests.

In the Methods section we added the following text:

Alternatively, the bootstrap script can be run from a Docker container published on DockerHub (https://cloud.docker.com/u/bionitio/repository/docker/bionitio/bionitio-boot):

```
$ docker run -it -v "$(pwd):/out" --rm bionitio/bionitio-boot \

-i python -n newproj -c BSD-3-Clause
```

Comment 9

(methods; choosing a language) do you have any way to recommend language selection for users? If they're truly new to all of these, maybe coming from a MATLAB background like many who learned to program through coursework, what guidance does Bionitio provide here? Is Python a general default, or just for this example? If it is, where is that justified? The caveat with providing 12 options is that a bit of hand holding may be required to guide the choice for much of your target audience.

Response

We agree with the reviewer that choice of programming language can be difficult for absolute beginners. It is difficult to get empirical evidence to support any language default (and for this reason Bionitio does not have a default language). However, the selection of implementation languages chosen was guided by the results reported in [13]. From an analysis of 1,720 bioinformatics repositories on GitHub they observed: "The main dataset contained a greater proportion of code written in interpreted or hybrid interpreted/compiled (such as Python) and dynamically typed languages" and "Our data support the intuition that Java, Python and R are more succinct than lower-level languages such as C and C++" Taking these observations together, Python appears to be reasonable starting language for beginners. To assist beginners with their choice of language we have updated the README (https://github.com/bionitio-team/bionitio) documentation for Bionitio to include:

> If you are new to programming, and do not know which programming language to use, then we recommend picking one of the high-level interpreted languages that are popular in Bioinformatics, such as Python or R. You may also need to seek advice from your peers about which language(s) are most appropriate for your purposes. We have tried to cover as many popular languages as possible, and apologise if your preference is not currently available. However, we also welcome new implementations of Bionitio in languages not already covered.

We have also added the following text to the manuscript:

> For users relatively new to programming, with no prior constraints on their choice of language, we recommend they choose a high-level interpreted language such as Python or R.

Comment 10

> can you justify the claims about it being an "excellent vehicle for education"? Any sort of case study or example from similar tools being effective, etc…

Response

We believe that Bionitio is fairly unique in its approach to templating best practices in Bioinformatics software development, and therefore it is unlikely that such an approach has been formally studied in the context of education practices, and unfortunately we are not aware of such resources (even beyond bioinformatics). However, as mentioned in our cover letter, we have used Bionitio as the basis for a popular (whole day) workshop hosted at the Australian Bioinformatics and Computational Biology Society (ABACBS) annual conference in 2018 (https://www.abacbs.org/conference2018) with ~50 paying attendees from around the country. We conducted a survey of the attendees to assess the quality and utility of the workshop. In response to the question "This was a useful workshop that enhanced my knowledge and skills" out of 18 respondents 94.44% agreed or strongly agreed. Given the success of the initial workshop, we ran another in May 2019, with 14 attendees. From formal feedback received from the second workshop, in response to the question "My overall impression is that this is a useful workshop that enhanced my knowledge and skills" we received a score of 4.8/5 from 11 respondents. We appreciate that this is anecdotal evidence and is not supported by a rigorous experiment and therefore we have not discussed the workshop feedback in the manuscript. However, we have reduced the strength of our claim in the manuscript by adding a qualifier:

> Additionally, by providing complete working examples in many different languages, Bionitio acts as a kind of "Rosetta Stone" and is therefore likely an excellent vehicle for comparative programming skills transfer.

We have also addressed Bionitio's role in training and education more thoroughly in the Conclusion as mentioned in our response to Comment 1 above.

Comment 11

> figure 1 text is barely readable, and boxes are odd relative sizes with a fair amount of wasted foreground (coloured) space. Colour doesn't seem to convey much information. I didn't find this figure particularly useful or instructive. I.e. I don't know any better how I would use bionitio, or what exactly it'll create (just that it draws from a boiler plate). Maybe repurpose this figure to be more of a "schematic" of what is contained within a bionitio-created-project (is there a more concise name for these?), and then a more streamlined version of what is currently here.

Response

We included this figure in the manuscript because it serves to visually represent how a new project is started by the bootstrap script. We have found that this has been a particular issue of confusion for new users, especially those who are unfamiliar with Git and GitHub. The colours represent the location of code, either in GitHub (yellow) or the local machine (purple). To streamline this figure, we have removed the grey background, replaced the external arrows with dashed lines. We will submit a high-resolution version in our resubmission, and include a resolution-independent SVG version as well.

Comment 12

> I felt that while the manuscript introduces a tool which is certainly of use to a community of scientific software developers, the focus of the paper is more based on the justification of which components are included in this tool, rather than the technical nature or efficacy of the contribution. With guides that exist and "best practices" that were even mentioned in the 10-simple-rules article, I believe the article would benefit from significant rewriting to be focused on the contributions of these authors and their tool, rather than an extended summary of what are commonly accepted as best practices for software development. While I acknowledge the novel and valuable contribution presented in this paper, I feel the manuscript does not highlight this contribution adequately.

Response

Thank you for recognising the utility of Bionitio. We believe this point is similar in to Comment 1 which we have addressed in detail above.

Comment 13

> One concern I have with making it easier for people to continue making their own tools is exactly to the point mentioned at one point in the manuscript, of "never repeat yourself." In my area of research, computational neuroscience, essentially every pipeline has been built handfuls of times, and the answers aren't particularly replicable across implementations. This of course raises a whole other set of issues in terms of the quality of software being produced, because even if we encourage developers to adopt existing tools where possible, if they don't adopt the same ones for the same tasks, how can we meaningfully compare their implementations? This ties in to the

> FAIR principles, which I was surprised not to see mention of in this work, as they are closely aligned with the aim of bionitio to my understanding. The missing piece in bionitio, of course, would then be that of publishing tools and ensuring the findability of software that people will make. This of course doesn't solve the issue, but at least enables the easier evaluation of various implementations towards the same end. As mentioned above, the Boutiques initiative (disclaimer: I am a co-lead on this project) makes efforts to make sure tools, once they exist, are able to be shared/consumed FAIR-ly, so could potentially be referenced in a discussion on this point. The paper of this tool is on Gigascience (https://doi.org/10.1093/gigascience/giy016) and a recent poster focusing on FAIR software workflows can be found here: https://doi.org/10.6084/m9.figshare.8143241.v2 . I would appreciate if the authors discussed this point, the obvious risk that their tool introduces into the field by virtue of increasing the accessibility of tool development, and how they propose their contribution is either worth this added risk or how they intend on enabling the evaluation of it.

Response

We agree with the reviewer that there can be a tendency in bioinformatics and scientific computing to reinvent the wheel, which can create challenges for replication and comparison. However, a key point is that the purpose of Bionitio is not to merely make it easier for people to continue making their own tools or make software development more accessible (though many other systems, such as IDEs, do this already), but rather to help people to make *better* tools. We anticipate that many uses of Bionitio will be for bespoke tools that are tailored to the specific needs of a research project - tools that do not already exist. In our experience there is considerable demand in Bioinformatics for the creation of this type of software. Even if these tools do not have a wide audience at the beginning, we still believe that there is considerable value to be gained from the use of good programming practices from the outset of the project, as discussed in the paper. We also agree with the reviewer that the FAIR principles are highly relevant and have made considerable changes to the manuscript to address this issue, as per our response to Comment 1. We thank the reviewer for bringing the Boutiques system to our attention. Command-line Interface and packaging standardisation efforts provided by Boutiques and similar systems such as CWL provide great utility in building usable and reproducible analyses. We commend and support these initiatives. We have added CWL tool wrapper support to Bionitio as discussed in our response to Comment 7 above.

Reviewer 2

Comment 14

> I wonder if the command line is the best starting point for inexperienced programmers? And if not starting with an IDE that provides similar templates would be better? The paper could therefore discuss how bionitio can be used from an IDE. I also wonder if the team plan to provide bionitio templates for some popular IDEs?

Response

We agree that providing support for IDE use may be of use to inexperienced programmers, however that would be challenging to do in a way that is portable across: all the (12) language implementations of Bionitio, the many popular IDEs that are currently in use, and the major operating systems. Considering these challenges, we decided to make Bionitio agnostic with respect to the programming environment employed by users. Users *can* use an IDE with Bionitio if they desire, but we do not plan to offer particular support for any of the many options available.

Comment 15

> are there some numbers that can document how much bionitio is used, and who the users are? Perhaps something from GitHub?

Response

We do not currently have information about how much Bionitio is used and who the users are. We use it ourselves on a regular basis, as do many of our colleagues. Many attendees of our workshops continue to use it for their own work. Our experience shows that it offers considerable utility for a wide audience.

Comment 16

> I tested bionitio-python in Ubuntu on Windows. It has Python2 as default, so by following the instructions I could install the code (with Python2), but not run it. However, by installing the code with pip3 I could use it as described in the documentation. But this solution may not be obvious for beginner programmers, so the documentation should take this into account.

Response

Only Python 3 is currently supported by Bionitio. Python 3 is at least 10 years old, and Python 2 is now officially considered a legacy system. New Python projects are encouraged to be written in Python 3. As the reviewer notes, it is unfortunate that some operating systems still offer Python 2 as a default. As suggested, we have updated the user documentation to make it clearer that Python 3 is required. Specifically, we have replaced Python with "Python 3" on the main README.md for Bionitio (https://github.com/bionitio-team/bionitio), and also added the text "Python 3 is required for this software." on the README.md for the Python implementation of Bionitio (https://github.com/bionitio-team/bionitio-python).

Comment 17

> I also found that the example program treated invalid fasta files (for example setup.py) as empty fasta files. I believe according to the documentation this should have resulted in an "invalid file" exit value instead of success (zero).

Response

We thank the reviewer for identifying this mistake. Most FASTA parsing libraries are very liberal in what they accept as valid input, and therefore do not raise errors on files that are not FASTA format. We had originally intended to return an exit status of 3 for input files not in FASTA format, but this is challenging to do when most standard libraries for parsing FASTA do not raise errors. Given this limitation, we have

decided to remove the exit status of 3 and have adjusted the documentation in the README file for each Bionitio implementation accordingly.

Comment 18

> In the documentation, C# was not mentioned as one of the available languages.
> The integration with GitHub, however, did not work. When we ran the script, a different command-line window was opened, asking for a GitHub password. When typing in the password, it was shown in clear text. Nothing happened when hitting the Enter-key. The GitHub-repository was not created.

Response

> We thank the reviewer for pointing out the omission of C# in the list of available languages. We have corrected this mistake in the README.md file for Bionitio (https://github.com/bionitio-team/bionitio/blob/master/README.md) and in the user documentation (https://github.com/bionitio-team/bionitio/wiki/2.-Set-up-a-project-with-bionitio).
>
> We have not been able to reproduce the error with GitHub integration on Windows. However, we have created an issue on our GitHub repository for Bionitio to look into this further, to see if it can be reproduced (https://github.com/bionitio-team/bionitio/issues/73).

Comment 19

> The C++ version requires a bit more manual configuration, since the `CMake`/`Make` install does not copy the built executable to a standard location (such as usr/local/bin). It also depends on the `seqan` library that has to be downloaded separately.

Response

> We agree with the reviewer that the C++ version requires more manual configuration than some of the other implementations of Bionitio. This is due to the limited nature of C++ software packaging systems. We require the user to download the Seqan source code from http://packages.seqan.de/seqan-library/seqan-library-2.1.1.tar.xz because we do not want to include that source code in the Bionitio repository. We have added an 'install' target for the build command now, so that the user can run 'make install' and have the executable copied into their desired location and have updated the documentation accordingly (https://github.com/bionitio-team/bionitio-cpp/blob/master/README.md). The default behaviour is to place the executable in the same directory where the 'make' command was executed. It is also likely that the added support for Docker will also help some users with this issue.

Reviewer 3

Comment 20

for Windows computer use case scenario, it only say to get Putty to login to Linux. I would suggest the authors create a Virtualbox or Docker container (both virtualizations solutions are as easy to install on Windows as you would install Skype), with some base ubuntu and some working examples included of project structure generation with their tool.

Response

Currently Bionitio is not directly supported on Windows. Our user guide suggested that Windows users might need to log into a Unix-like system to try it out. We have added the reviewer's suggestion that they may also like to consider the use of a virtualisation system such as VirtualBox (https://github.com/bionitio-team/bionitio/wiki/1.-Set-up-your-computer). We agree with the reviewer that the addition of Docker container support will be a benefit to Windows users.

Comment 21

It would be great to have a little discussion how it can help beginner developers, for example can it be used in an educational setting when setting up projects for a bioinformatics class. It could be also discussed how advanced developers can use that to get project structure initiated fast (I am thinking Ruby on Rails or Django project initiation). What are the additional benefits for advanced developers ?

Response

Yes, Bionitio is well suited to being used in an educational setting. We have developed and delivered workshops on best practices in bioinformatics software development based on Bionitio. We have added a new section to the manuscript on its role in education and training (see our previous response to Comment 1 above). We agree that Bionitio can also help advanced users get project structure initiated quickly. Indeed, we count ourselves as advanced users, and we regularly use Bionitio for this purpose. We believe that the use-case suggested by the reviewer (Ruby on Rails or Django project) is better solved by the Cookiecutter project, mentioned as related work in the Background section where we note "Cookiecutter provides a more general-purpose templating system that is best suited to starting new software systems in specific programming languages, such as the instantiation of web applications based on particular web framework libraries."

Additional changes to the manuscript

The following small improvements were made to the manuscript.

1) Affiliation updated for one author:

Melbourne Genomics Health Alliance, Walter and Eliza Hall Institute, Parkville, Victoria, Australia.

2) A sentence in the Abstract was reworded to improve expression:

Old sentence:

On the other hand, the barrier to entry in bioinformatics software development is high for beginners, especially if they want to adopt good programming practices.

Replacement:

In particular, for beginners, the barrier to entry in bioinformatics software development is high, especially if they want to adopt good programming practices.

3) Text added to describe the optional --minlen command line argument in the Design and Implementation section:

An optional command line argument --minlen can be supplied, causing the program to ignore sequences with length strictly less than the given value.

4) Paragraph slightly reworded in the Design and Implementation section to improve expression:

Old paragraph:

In the remainder of this section we outline the main features incorporated into Bionitio's prototypical tool that facilitate good programming practices and why they are important. In the following section we demonstrate by example how Bionitio can be used to create a new software project.

New paragraph with changes noted in red:

In the remainder of this section we outline the main features incorporated into Bionitio's prototypical tool that facilitate good programming practices, and, where possible, relate them to the relevant recommendations in the literature. In the section afterwards we demonstrate by example how Bionitio can be used to create a new software project.

5) Replaced "in name of" with "for the sake of" in the Conclusion to improve expression.

6) We capitalised "Cookiecutter" in the Background section.

7) We shifted the sentence "(the `$` sign indicates the command line prompt):" earlier in the text, to cover the first use of the $ notation.

8) We improved the wording of the first sentence in the sub-section "Standardised software packaging and containerisation" (and added an additional reference): "The installation process can be one of the most cumbersome and frustrating parts of using bioinformatics software, because many tools do not provide much assistance to the user [14], and complex dependency chains can clash with local settings [10]."

9) Fixed the pluralisation in the Revision control sub-section: "implementations"
