# Supplementary material for: Bionitio: demonstrating and facilitating best practices for bioinformatics command-line software
Source: Gigascience. 2019 Sep 23;8(9):giz109. doi: 10.1093/gigascience/giz109 (PMC6755254; doi:10.1093/gigascience/giz109)
Supplement: giz109_GIGA-D-19-00145_Revision_2 [file giz109_giga-d-19-00145_revision_2.pdf]

GigaScience

Bionitio: demonstrating and facilitating best practices for bioinformatics command-line software

--Manuscript Draft--

**Manuscript Number:**	GIGA-D-19-00145R2
**Full Title:**	Bionitio: demonstrating and facilitating best practices for bioinformatics command-line software
**Article Type:**	Technical Note
**Funding Information:**	
**Abstract:**	Background<br><br>Bioinformatics software tools are often created ad hoc, frequently by people without extensive training in software development. In particular, for beginners, the barrier to entry in bioinformatics software development is high, especially if they want to adopt good programming practices. Even experienced developers do not always follow best practices in all the code they develop. A consequence of this is the proliferation of poorer-quality bioinformatics software, leading to limited scalability and inefficient use of resources; lack of reproducibility, usability, adaptability and interoperability; and erroneous or inaccurate results.<br><br>Findings<br><br>In response to this problem we have developed Bionitio, a tool that automates the process of starting new bioinformatics software projects following recommended best-practices. With a single command, the user can create a new well-structured project in one of twelve programming languages. The resulting software is functional, carrying out a prototypical bioinformatics task, and thus serves as both a working example and a template for building new tools. Key features include command-line argument parsing, error handling, progress logging, defined exit status values, a test suite, a version number, standardised building and packaging, user documentation, code documentation, a standard open-source software license, software revision control, and containerisation.<br><br>Conclusions<br><br>Bionitio serves as a learning aid for beginner-to-intermediate bioinformatics programmers and provides an excellent starting point for new projects. This helps developers adopt good programming practices from the beginning of a project and encourages high-quality tools to be developed more rapidly. This also benefits users of the tools because they are more easily installed and consistent in their usage. Bionitio is released as open source software under the MIT License, and is available at https://github.com/bionitio-team/bionitio.
**Corresponding Author:**	Bernie Pope, Ph.D.<br>The University of Melbourne, Australia<br>AUSTRALIA
**Corresponding Author Secondary Information:**	
**Corresponding Author's Institution:**	The University of Melbourne, Australia
**Corresponding Author's Secondary Institution:**	
**First Author:**	Peter Georgeson
**First Author Secondary Information:**	
**Order of Authors:**	Peter Georgeson
	Anna Syme

Powered by Editorial Manager® and ProduXion Manager® from Aries Systems Corporation

	Clare Sloggett
	Jessica Chung
	Harriet Dashnow
	Michael Milton
	Andrew Lonsdale
	David Powell
	Torsten Seemann
	Bernard Pope, Ph.D.
**Order of Authors Secondary Information:**	
**Response to Reviewers:**	Dear GigaScience Editors,<br><br>Thank you for accepting our manuscript for publication.<br><br>In response to the comments from the Editor and reviewer we have made the following small changes to the manuscript:<br><br>1) We have cited the Boutiques software, as recommended by the reviewer.<br>2) We have added a link to the archival snapshot of your code in gigaDB.<br>3) We have made minor corrections for typos, grammar, format, and have removed any tracking and red color that was made to highlight changes for the reviewers.<br><br>Kind regards,<br>Bernard Pope
**Additional Information:**	
**Question**	**Response**
Are you submitting this manuscript to a special series or article collection?	No
**Experimental design and statistics**<br><br>Full details of the experimental design and statistical methods used should be given in the Methods section, as detailed in our Minimum Standards Reporting Checklist. Information essential to interpreting the data presented should be made available in the figure legends.<br><br>Have you included all the information requested in your manuscript?	Yes
**Resources**<br><br>A description of all resources used, including antibodies, cell lines, animals and software tools, with enough information to allow them to be uniquely identified, should be included in the	Yes

Methods section. Authors are strongly encouraged to cite Research Resource Identifiers (RRIDs) for antibodies, model organisms and tools, where possible.<br><br>Have you included the information requested as detailed in our Minimum Standards Reporting Checklist?	
**Availability of data and materials**<br><br>All datasets and code on which the conclusions of the paper rely must be either included in your submission or deposited in publicly available repositories (where available and ethically appropriate), referencing such data using a unique identifier in the references and in the “Availability of Data and Materials” section of your manuscript.<br><br>Have you have met the above requirement as detailed in our Minimum Standards Reporting Checklist?	Yes

Powered by Editorial Manager® and ProduXion Manager® from Aries Systems Corporation

Manuscript

Click here to access/download;Manuscript;bionitio_manuscript_final.docx

Click here to view linked References

Bionitio: demonstrating and facilitating best practices for bioinformatics command-line software

Peter Georgeson: Melbourne Bioinformatics, The University of Melbourne, Melbourne, Victoria, Australia. Department of Clinical Pathology, The University of Melbourne, Australia, peter.georgeson@unimelb.edu.au

Anna Syme: Melbourne Bioinformatics, The University of Melbourne, Melbourne, Victoria, Australia. Royal Botanic Gardens Melbourne, Victoria, Australia. anna.syme@rbg.vic.gov.au

Clare Sloggett: Melbourne Bioinformatics, The University of Melbourne, Melbourne, Victoria, Australia. sloc@unimelb.edu.au

Jessica Chung: Melbourne Bioinformatics, The University of Melbourne, Melbourne, Victoria, Australia. jchung@unimelb.edu.au

Harriet Dashnow: Bioinformatics, Murdoch Children's Research Institute, Royal Children's Hospital, Parkville, Victoria, Australia and School of BioSciences, The University of Melbourne, Melbourne, Victoria, Australia. harriet.dashnow@mcri.edu.au

Michael Milton: Melbourne Bioinformatics, The University of Melbourne, Melbourne, Victoria, Australia, Melbourne Genomics Health Alliance, Walter and Eliza Hall Institute, Parkville, Victoria, Australia. michael.milton@unimelb.edu.au

Andrew Lonsdale: ARC Centre of Excellence in Plant Cell Walls, School of BioSciences, The University of Melbourne, Parkville, Victoria, Australia and Bioinformatics, Murdoch Children's Research Institute, Royal Children's Hospital, Parkville, Victoria, Australia. andrew.lonsdale@lonsbio.com.au

David Powell: Monash Bioinformatics Platform, Biomedicine Discovery Institute, Faculty of Medicine, Nursing and Health Sciences, Monash University, Clayton, Victoria, Australia. david.powell@monash.edu

Torsten Seemann: Melbourne Bioinformatics, The University of Melbourne, Melbourne, Victoria, Australia; Department of Microbiology and Immunology, Doherty Institute for Infection and Immunity, The University of Melbourne, Melbourne, Victoria, Australia. t.seemann@unimelb.edu.au

Bernard Pope: Melbourne Bioinformatics, The University of Melbourne, Melbourne, Victoria, Australia. Department of Clinical Pathology, The University of Melbourne, Australia. Department of Medicine, Central Clinical School, Monash University, Australia. bjpope@unimelb.edu.au. (* Corresponding author)

Abstract

Background

Bioinformatics software tools are often created *ad hoc*, frequently by people without extensive training in software development. In particular, for beginners, the barrier to entry in bioinformatics software development is high, especially if they want to adopt good programming practices. Even experienced developers do not always follow best practices in all the code they develop. A consequence of this is the proliferation of poorer-quality bioinformatics software, leading to limited scalability and inefficient use of resources; lack of reproducibility, usability, adaptability and interoperability; and erroneous or inaccurate results.

Findings

In response to this problem we have developed Bionitio, a tool that automates the process of starting new bioinformatics software projects following recommended best-practices. With a single command, the user can create a new well-structured project in one of twelve programming languages. The resulting software is functional, carrying out a prototypical bioinformatics task, and thus serves as both a working example and a template for building new tools. Key features include command-line argument parsing, error handling, progress logging, defined exit status values, a test suite, a version number, standardised building and packaging, user documentation, code documentation, a standard open-source software license, software revision control, and containerisation.

Conclusions

Bionitio serves as a learning aid for beginner-to-intermediate bioinformatics programmers and provides an excellent starting point for new projects. This helps developers adopt good

programming practices from the beginning of a project and encourages high-quality tools to be developed more rapidly. This also benefits users of the tools because they are more easily installed and consistent in their usage. Bionitio is released as open source software under the MIT License, and is available at https://github.com/bionitio-team/bionitio.

Keywords

bioinformatics, software development, best practices, training

Findings

Background

Software development is a central part of Bioinformatics, spanning a wide gamut of activities including data transformation, scripting, workflows, statistical analysis, data visualisation, and the implementation of core analytical algorithms. However, despite the critical and far-reaching nature of this work, there is a high degree of variability in the quality of bioinformatics software tools being developed, reflecting a broader trend across all scientific disciplines [1–3].

A common approach to defining software quality is to consider how well it meets its requirements. These can be *functional* — identifying what the software should do, and *non-functional* — identifying how it should work. Given the results-driven nature of research, the functional aspects of scientific programs (e.g. whether expected inputs produce expected outputs) are heavily emphasised at the expense of the non-functional ones (e.g. usability, maintainability, interoperability, efficiency) [4]. Additionally, the highly complex and evolving nature of scientific software can make software requirements specifications (SRSs) infeasible, and therefore they are rarely defined in practice [4,5].

The underlying causes of poor bioinformatics software quality are multifaceted, however two important factors have been highlighted in the literature: 1) the lack of software engineering training amongst bioinformaticians [2,3,6–11]; and 2) the fact that research groups have limited time and money to spend on software quality assurance [10,12–15]. As a result many bad practices are recurrently observed in the field. Lack of code documentation and user support makes tools hard to install, understand and use. Limited or non-existent testing can result in unreliable and buggy behaviour. A high-degree of coupling with the local computing environment and software dependencies impedes portability. The consequences of poor quality software can have a significant impact on scientific outcomes. Substantial amounts of users' time can be wasted in trying to get programs to work, scientific methods can be difficult to reproduce, and in the worst-case, scientific results can be invalid due to program errors or incorrect usage [3,7,8,10,12,13,16,17].

The above-mentioned problems are well known and have prompted remedial action in a number of areas. Activities to increase software development training amongst scientists are under way, the most notable examples being the highly successful Software Carpentry and Data Carpentry workshops [2,3]. Additionally, there are many useful recommendations in the literature offering practical advice for beginners [9,12,16,18] including specific advice for biologists new to programming [19]. Significant efforts have also been made in producing software package collections where best-practice guidelines and curation provide minimum standards of software quality, such as Bioconductor for R [20], and Bioconda for bioinformatics command-line tools [21], to name two prominent examples. Operating system virtualisation services, such as Docker [22], and workflow specification languages, such as the Common Workflow Language (CWL) [23], have improved portability and reproducibility of tools and pipelines [12,24–26], while systems such as Boutiques [27] have enhanced the findability of tools by facilitating the publication of persistent metadata. Increasing the resources available for scientific software development remains a complex challenge. The

Software Sustainability Institute in the UK demonstrates one successful model where pooled research funding enables the provision of consultancy, training and advocacy for scientific software development on a national level [28].

In this work we adopt a pragmatic approach to improving bioinformatics software quality that is summarised by Rule 7 in Carey and Papin's *Ten simple rules for biologists learning to program*: "develop good habits early on" [19]. The idea is that new bioinformatics tools should be started by copying and modifying a well-written existing example. This allows bioinformaticians to get started quickly on solving the crux of their problem, but also ensures that all the ingredients of good programming style and functionality are present from the beginning. Based on this concept we have developed a tool called Bionitio that automates the process of starting new bioinformatics software projects with recommended software best-practices built-in. With a single command the user can create a new well-structured project in one of (currently) twelve programming languages. The resulting software is functional, carrying out a prototypical bioinformatics task, and thus serves as both a working example and a template for building new tools. It is expected that users will incrementally modify this program to ultimately satisfy the requirements of their task at hand. The key point is that they are building on solid foundations, and because a lot of the mundane-but-important boilerplate is provided by Bionitio, there are fewer barriers to adopting good practices from the start. Specifically, every new Bionitio-created project includes command-line argument parsing, error handling, progress logging, defined exit status values, a test suite, a version number, standardised building and packaging, user documentation, code documentation, a standard open-source software license, software revision control, containerisation with Docker, and a CWL wrapper. In this paper we describe the design and implementation of Bionitio and demonstrate how it can be used to quickly start new bioinformatics projects.

The closest related work to Bionitio is the Cookiecutter project [29]. It also takes advantage of the templating approach for starting new software projects, but it is targeted at a different audience. Cookiecutter provides a more general-purpose templating system that is best suited to starting new software systems in *specific* programming languages, such as the instantiation of web applications based on particular web framework libraries. Conversely, Bionitio provides many instances of the same prototypical bioinformatics tool implemented in *different* programming languages. While Bionitio could theoretically be implemented on top of a system such as Cookiecutter, we believe that the extra complexity is not warranted and would be a barrier to understanding for our target audience.

Design and Implementation

Bionitio is designed around two components.

The first component is a prototypical bioinformatics tool that has been re-implemented in (currently) twelve different programming languages. Each implementation of the tool carries out exactly the same task, and each is stored in its own repository on GitHub underneath the bionitio-team project. For example, the Python 3 and C++ implementations are found at the following GitHub URLs respectively:

https://github.com/bionitio-team/bionitio-python
https://github.com/bionitio-team/bionitio-cpp

Each of the repositories acts as a self-contained exemplar of how to implement the prototypical tool in the given programming language, carrying out good programming practices (e.g. command-line argument parsing) in a language-idiomatic way.

The second component is a "bootstrap" script that automates the process of creating a new software project based on one of the language-specific repositories. With a single invocation of the bootstrap script the user can quickly start a new project; all they need to do is specify a new project name and the programming language to use (the `$` sign indicates the command-line prompt):

```
$ bionitio-boot.sh -n newproj -i python
```

The example above creates a new local repository called "newproj" on the user's computer by cloning and then renaming the bionitio-python repository. Optionally, the user can also specify their GitHub username, which will cause the bootstrap script to create and populate a remote repository on GitHub for the new project. The repository comes with a test-suite, allowing continuous integration testing to easily be enabled via GitHub's integration with Travis CI [30]. The overall process carried out by the bootstrap script is illustrated in Figure 1.

Figure 1. Overview of the automated process for creating new projects performed by the Bionitio bootstrap script.

The prototypical bioinformatics tool is intended to be easy to understand and modify. Therefore it has only minimal functionality; just enough to demonstrate all the key features of a real bioinformatics command-line program without becoming distracted by unnecessary complexity. In essence, the tool streams input from one or more FASTA files, computes several simple statistics about each file, and prints a tabulated summary of results on standard output. For example, the command below illustrates the behaviour of the tool on a single input FASTA file called "file1.fa":

```
$ bionitio file1.fa
```

```
FILENAME  NUMSEQ  TOTAL    MIN  AVG  MAX
file1.fa  5264    3801855  31   722  53540
```

The output is in tab-delimited format, consisting of a header row, followed by one or more rows of data, one for each input file. Each data row contains the name of the input file, followed by the total number of sequences in the file (NUMSEQ), the sum of the length of all the sequences in the file (TOTAL), followed by the minimum (MIN), average (AVG), and maximum (MAX) sequence lengths encountered in the file. An optional command-line argument --minlen can be supplied, causing the program to ignore sequences with length strictly less than the given value.

Each implementation is self-contained and ready to be installed and executed. Consequently, Bionitio is an excellent resource for programmer training. However, the main intended use-case is that Bionitio will be used as the starting point for new projects and we expect users to rewrite parts of it to carry out their own desired functionality. Given that much of the boilerplate is already provided, modifying the program should be significantly easier than starting from scratch.

The twelve current implementation languages were chosen to represent the most commonly used languages in bioinformatics [17] (C, C++, Java, Javascript, Perl, Python, R and Ruby) but also to provide examples in up-and-coming languages and paradigms (C#, Clojure, Haskell and Rust). The fact that each instance implements the same prototypical tool provides important consistency amongst the different instances. It means that they all have common functionality, they can be easily compared, they can share the same test suite, their user documentation in the form of a README file can be templated, and the inclusion of new programming language implementations is straightforward. Over time we hope that new language implementations will be contributed by the community.

All the components of Bionitio are released under the terms of the MIT license, however we explicitly grant users permission to choose their own license for derivative works. The bootstrap script optionally allows the user to choose one of several standard open source licences for newly created projects (Apache-2.0, BSD-2-Clause, BSD-3-Clause, GPL-2.0, GPL-3.0 and MIT). If no license is specified, the MIT is chosen as the default. The terms of the license are copied into the LICENSE file in the top level of the repository, and all references to the license in source files are updated accordingly.

The bootstrap script also accepts optional author name and email address arguments which, if supplied, are inserted into the source code and documentation files at appropriate places. Newly created projects are committed to fresh Git [31] repositories. All instances of the word "bionitio" are replaced with the new project name, including in file paths and file contents, and all files are checked into a new git repository with a pristine commit history.

In the remainder of this section we outline the main features incorporated into Bionitio's prototypical tool that facilitate good programming practices, and, where possible, relate them to the relevant recommendations in the literature. In the following section we demonstrate by example how Bionitio can be used to create a new software project.

Table 1 to appear here [See Additional file 1].

Command-line argument parsing

We provide a standard command-line interface that follows modern Unix conventions [32,33], including providing arguments for help (--help) and the program version (--version) [18,34], and provision of single-dash notation for short argument names and double-dash notation for long argument names. Most importantly, the help argument causes the program to display usage information, including a description of each argument and its expected

parameters. Where possible we use standard library code for implementing command-line argument parsing (Table 1), which tends to simplify the process of adding new arguments and ensures that usage documentation is generated.

Input and output conventions and progress logging

Bioinformatics tools are often strung together in pipelines. A common Unix paradigm is that each tool should "expect the output of every program to become the input to another, as yet unknown, program" [35]. Consequently, the tool can take input from one or more files or from the standard input device (stdin), which may be piped from the output of another program. Similarly, output is written to the standard output device (stdout) in a tab-delimited format. Additionally, we ensure that error messages are always written to the standard error device (stderr) [18]).

We provide an optional progress logging facility (--log), providing useful metadata about a computation that can aid debugging and provenance [11]. Progress logging messages are written to a specified output file. The log file includes the command line used to execute the program, and entries indicating which files have been processed so far. Events in the log file are annotated with their date and time of occurrence. Where possible we use standard library code for the provision of logging services (Table 1), as these easily facilitate advanced features such as timestamping of log messages, log file roll-over, support for concurrency, and different levels of logging output (e.g. messages, warnings, errors, *etcetera*).

Library code for parsing common bioinformatics file formats

There are several tasks in bioinformatics that are common across analyses. For example, many tools will need to parse sequence files in FASTA format. Rather than re-write code for

this, it is better to use existing libraries. "Don’t Repeat Yourself" is a maxim that can be applied at multiple levels when programming [11,12,36]. Millions of lines of high-quality open source software are freely available on the web. It is typically better to find an established library or package that solves a problem than to attempt to write one's own routines for well-established problems [3] and this also improves reusability [10]. We demonstrate this principle by using existing bioinformatics library code to parse the input files (Table 1). This also allows Bionitio to demonstrate how non-standard library dependencies can be specified in the software package description, such as the "setup.py" file for Python that specifies a dependency on the biopython [37] library.

Defined exit status values

Processes on most operating systems return an integer exit status code upon termination. The Unix convention is to use zero for success and non-zero for error. Exit status values provide essential information about the behaviour of executed programs and are relied upon when programs are called within larger systems, such as bioinformatics pipelines. Such pipelines can become large and complex and can run for long periods of time, therefore the likelihood of errors is high. Improper indication of success or failure can have significant consequences for such systems. For example, erroneous reporting of exit status zero, for a computation that actually failed, can cause a pipeline to continue processing on incomplete results, yielding unpredictable behaviour, or worse, silent errors. Non-zero exit status values can also provide useful debugging information by distinguishing different classes of errors. Bionitio demonstrates good programming style by defining the exit status values as constants, and provides well-defined exit points in the program, and documents the meaning of the status values in the README file.

A test suite including unit tests, integration tests, and continuous integration

Software testing enables us to verify that the various components of the program work as expected, it allows us to modify the codebase while maintaining established functionality, and provides examples that demonstrate how to use the software along with its expected behaviour [16].

Bionitio includes examples of both unit tests and integration tests. A unit test runs a single method in isolation and enables the verification that each method in the implementation behaves as expected without concern for its extended environment. Where possible we use unit testing library frameworks appropriate for each programming language because they offer significant extended functionality over hand-written tests, and can facilitate better output reporting (Table 1). Integration tests ensure that the program behaves correctly as an entire entity, with all the components working together. Given that all implementations of Bionitio are expected to behave in the same way, they all share the same underlying testing data and automated integration-testing shell script. The README file for the project shows how the user can run a simple test to ensure that the program is working as expected, which increases their confidence that it was installed correctly [12].

Continuous integration is a software development practice that requires all changes to a software project's code base to be integrated, compiled and tested as changes are made. Travis is an online provider of continuous integration testing that enables automatic execution of tests whenever changes are committed to a source repository, and is currently available free to all GitHub users. This benefits software development by enabling any introduced problems to be identified faster [38], and avoids the introduction of breaking changes into the code. Each Bionitio implementation includes all the necessary Travis configuration files and demonstrates how continuous integration can be used to run both the unit and integration tests at each commit to the GitHub repository. The Bionitio wiki on GitHub contains detailed instructions about how to enable Travis for newly created projects. The README file also includes the URL to show the status badge for Travis testing,

providing a quick way for users to see the status of continuous integration testing (for example, a green icon badge showing successful "build passing").

Version number

Version numbers allow users to track the provenance of their work [11,12,18]. This is particularly important in Science where reproducibility is a primary concern. Bionitio comes with a clearly defined version number which is defined as a constant in a single place in the source code, which can be displayed to the user of the program via the --version command-line argument. We do not prescribe a particular versioning scheme to use (e.g. Semantic Versioning [39]), rather we prefer to let the user decide on the most appropriate mechanism for their work. Our main objective is that a version number is defined, that it can be easily discovered by the user, and that it is easy to update and modify in a single place in the program source code.

Standardised software packaging and containerisation

The installation process can be one of the most cumbersome and frustrating parts of using bioinformatics software, because many tools do not provide much assistance to the user [10], and complex dependency chains can clash with local settings [25]. Difficult to install software reduces reproducibility, is less likely to be used, and can cause problems with reliability due to differences between the developer and user computing environments. These problems can be addressed by using standard build tools and software packaging systems [12]. Such systems can automate the process of ensuring that correct and complete versions of software dependencies are installed [18], and by following conventional practice, they allow tools to integrate with the broader software ecosystem and follow the principle of least surprise [40]. Bionitio does this by adopting the idiomatic package and installation

mechanisms for each implementation language. For example in Python we use Pip, in C we use GNU autotools and make, and in C++ we use CMake. A full list of the building and packaging systems used in each implementation is provided in Table 1.

Standard packaging also helps with containerisation, which is becoming increasingly useful in bioinformatics [41]. Docker containers are a popular implementation of this concept, where the underlying operating system is virtualised and packaged alongside tools and their dependencies. This makes it easy to install "containerised" software on any platform that supports Docker, and facilitates reproducibility by enabling the exact same software build to be used on every system. Each Bionitio implementation comes with a "Dockerfile" that encodes all the necessary information needed to create a containerised version of the tool. As an added benefit, the Docker container is used in Travis continuous integration testing, which both simplifies the use of Travis and also enables the functionality of the container itself to be included in the tests.

A standard open-source software license

When software is distributed without a license it is generally interpreted to mean that no permission has been granted from the creators of the software to use, modify, or share it. This is counterproductive to adoption. A standard open-source license provides minimum fuss for users and increases the chances that software will be widely used [11], partly because it removes barriers to widespread access, and partly because it encourages transparency, reuse and collaboration [16]. Many license options are available [42]. As mentioned above, new projects started with Bionitio use the MIT license by default, but the user can choose from a number of standard options. The terms of the license are copied into the LICENSE file in the top level of the repository, and the name of the license is indicated prominently in the README file, and in source code files.

Documentation

Software documentation broadly falls into two categories: user documentation that explains how to install and use the code, and developer documentation that explains how the program is designed and intended to work. For the intended use case of Bionitio we believe it is important to strike a balance between the extensiveness of documentation and the effort required to maintain it. Therefore we adopt pragmatic recommendations from the literature that offer a good compromise between cost and functionality.

For user documentation we provide two critical components: a README file that appears at the top level of the repository, and comprehensive command-line usage output via the --help argument [18,34,38] as discussed above. The README file includes a program description, dependencies, installation instructions, inputs and outputs, example usage, and licensing information [12,43]. To ease the burden of adding new implementations of Bionitio, and to ensure consistency across current implementations, we build each README file from a template, such that common parts of the documentation are shared, and language-specific details (such as installation instructions) can be instantiated as needed.

Good developer documentation tries to explain the reasoning behind the code rather than recapitulating its operations in text [3], and can improve code readability, usability and debugging [34]. In Bionitio we adopt the following conventions in each implementation. Every source code file begins with header documentation that contains at least the following information: the name of the module, a brief description of its purpose, copyright information (author names and date of creation), license information, a maintainer email address, and a concise summary of the main components and processes undertaken in the module. Author names, creation dates, license name and maintainer email address can be automatically populated by the bootstrap script. Every non-trivial component of code (such as type definitions and procedures) are accompanied by a brief description of the purpose of the

component, plus descriptions of the arguments and results of methods, including any conditions that are assumed to uphold.

Revision control

Software revision control provides a systematic way to manage software updates, allowing multiple branches of development to be maintained in parallel, and provides a critical means of coordinating groups of developers [11,12,38]. Modern revision control systems such as Git [31] provide flexible and scalable modes of collaboration, supporting individual programmers all the way up to large — and potentially geographically distributed — teams. The collaborative advantages of Git are complemented by the GitHub code hosting web application [44], currently the most popular repository for bioinformatics code [17]. GitHub adds issue tracking, documentation publishing, lightweight release management, integration with external tools such as continuous integration testing, and perhaps most importantly, an easy-to-use web interface for source browsing and discovery. Bionitio takes advantage of Git and GitHub in two ways. Firstly, the Bionitio project itself is hosted on GitHub, including each of the twelve language-specific implementations of our prototypical bioinformatics tool. The bootstrap script creates new projects by cloning from GitHub, and therefore GitHub acts as our web-accessible content management system. Where possible, common features amongst the implementations, such as testing data, are shared via Git submodules, avoiding repetition. Secondly, the bootstrap script makes it easy for users to create new GitHub-hosted projects by optionally automating the initialisation and population of new repositories via the GitHub API. This saves the user’s time, encourages the use of revision control from the start of the project, and facilitates sharing the code with collaborators.

Recommended programming conventions

Each implementation of Bionitio aims to follow the programming conventions of the implementation language. This includes the adoption of standard tools and libraries as well as adhering to programming style guidelines, such as PEP 8 in Python. By following these practices we enhance integration with the language ecosystem, avoid common pitfalls, and encourage contributions from external developers [38,45]. Where possible, we have adopted automated code formatting tools to ensure that we adhere to recommended style, and static analysis tools to identify likely infelicities and possible sources of error. A full list of the code formatting and static analysis tools used in each implementation is provided in Table 1.

CWL tool wrapper

Bioinformatics pipelines — where multiple tools are chained together to perform an overall analysis — create further challenges for reproducible science. This has motivated the creation of pipeline frameworks that allow the logic of such computations to be abstracted from the details of how they are executed. An emerging standard in this area is the Common Workflow Language (CWL) that is supported by several popular workflow engines. CWL comprises two declarative sub-languages: workflow descriptions, that define data flow patterns between pipeline stages; and command-line tool descriptions, that define the interfaces of tools in a platform independent manner. Each Bionitio implementation provides a CWL tool description "bionitio.cwl", that facilitates its incorporation into CWL pipelines, and takes advantage of CWL's support for invoking programs within Docker containers.

Methods

In this section we demonstrate how to create a new bioinformatics software project using the Bionitio bootstrap script. In order to follow this process the user requires a GitHub account, and installation of Git on their local computer.

Step 1: choose a programming language, project name, and software license

The Bionitio prototypical bioinformatics tool is currently implemented in twelve programming languages: C, C++, C#, Clojure, Java, Javascript, Haskell, Perl5, Python, R, Ruby, or Rust. The user must choose which of these languages they want to use for their new project. For users relatively new to programming, with no prior constraints on their choice of language, we recommend they choose a high level interpreted language such as Python or R. The user must also choose a new name for their project. Optionally, the user may also choose an open source license for their code. The current available options are Apache-2.0, BSD-2-Clause, BSD-3-Clause, GPL-2.0, GPL-3.0 and MIT. If no license is specified, the MIT license is selected by default. In this example we will assume that Python is chosen as the implementation language, the project name is "newproj", and the BSD-3-Clause license is desired.

Step 2: run the bootstrap script to create a new software repository

The Bionitio bootstrap script is a BASH shell script that automates the process of creating new projects. In principle, if Bionitio is already installed on the user's computer, then the bootstrap script can be run like so:

```
$ bionitio-boot.sh -i python -n newproj -c BSD-3-Clause
```

A user may find it inconvenient to have Bionitio installed just to run the bootstrap script, therefore they may instead prefer to use Curl [46] to simplify the process, by downloading the script directly from GitHub before running it locally:

```
$ URL=https://raw.githubusercontent.com/\
bionitio-team/bionitio/master/boot/bionitio-boot.sh
```

```
$ curl -sSf $URL | bash -s -- -i python -n newproj -c BSD-3-Clause
```

Alternatively, the bootstrap script can be run from a Docker container published on DockerHub (https://cloud.docker.com/u/bionitio/repository/docker/bionitio/bionitio-boot):

```
$ docker run -it -v "$(pwd):/out" --rm bionitio/bionitio-boot \
-i python -n newproj -c BSD-3-Clause
```

The user may optionally specify an author name and email address, which will be substituted for placeholders in the source code and documentation at appropriate places:

```
$ bionitio-boot.sh -i python -n newproj -c BSD-3-Clause \
-a "Example Author" -e example.email@institute.org
```

Finally, the user may specify a GitHub username. In this circumstance the bootstrap script will create a new remote repository under the specified project name on GitHub and push the project to that repository:

```
$ bionitio-boot.sh -i python -n newproj -c BSD-3-Clause \
-a "Example Author" -e example.email@institute.org -g example_github_user
```

Step 3: run the test suite, and optionally setup continuous integration testing

Each new repository created by the bootstrap script contains a testing directory called "functional_tests". Within that directory is an automated testing shell script called (in this example) "newproj-test.sh" and a sub-directory of test data and corresponding expected outputs. The test script can be run like so:

```
$ newproj-test.sh -p newproj -d test_data
```

The test script reports how many tests passed and failed, and an optional -v (to enable verbose mode) will cause it to report more details about each test case that is run. Obviously, the test cases are specific to the expected behaviour of the prototypical bioinformatics tool implemented by Bionitio. It is expected that the user will replace these tests to suit the requirements of their new project. Despite this, the user will benefit from much of the testing infrastructure provided by the script.

If the user has created a remote repository for their project on GitHub, they can quickly enable continuous integration testing via Travis CI. Each new project created by Bionitio includes the necessary Travis configuration files that are needed to install the prototypical bioinformatics tool and run the integration and unit test scripts.

From this point onwards we expect that the user will go on to modify the program in order to carry out their intended task. This includes changing the code of the program itself, updating library dependencies, and importantly, adding appropriate test cases.

Conclusions

Software development is a complex task, involving many concepts and processes that can be daunting for beginners. Many bioinformaticians are not trained in software engineering, and research-oriented projects have limited budgets for quality assurance. The results-driven focus of science means that many important non-functional software requirements are often overlooked. Unfortunately these factors mean that shortcuts are often taken for the sake of making something "that works", leading to a proliferation of lower-quality bioinformatics tools.

Bionitio takes a pragmatic approach to addressing this problem. Our ambition is to help beginner and intermediate bioinformaticians develop good habits early on. We aim to achieve this by automating much of the drudgery involved in setting up new projects by providing a simple working example that has the necessary boilerplate in place. By providing a fast and simple way to start new projects from solid foundations we believe that good practices are more likely to be adopted.

The challenges faced by the bioinformatics and science communities in building better quality software are well known, and there is no shortage of practical recommendations to be found in the literature. These guidelines are undoubtedly useful to beginners, however, we believe they fall short in two ways. First, they are spread over multiple manuscripts that only partially overlap in their recommendations, therefore some level of consolidation is needed. Second, they are static artefacts that point to good practices but do not remove the considerable burden of applying them in real code. These two observations motivated the creation of Bionitio, both as a way of collecting commonly recommended best practices, and as a way of demonstrating and facilitating their use. Therefore a significant contribution of our work is to build a tool that can both illustrate best practices by example but also make it easy to use them in new projects. In this sense Bionitio takes a much more active role in the dissemination and compliance with these principles.

Role in education and training

In very recent work Tractenberg *et al* have developed a Mastery Rubric for Bioinformatics with the goal of better defining skills development and competencies in the discipline [47]. In this framework, competency in computational methods ranges through five levels, from novice (stage 1) to independent bioinformatics practitioner (stage 5). One of the goals of Bionitio is to support education and training for advancing bioinformaticians from stage 3 — learning best practices in programming, and writing basic code — to stage 5 — developing

new software that is useful, efficient, standardized, well-documented and reproducible. As an example of this application, Bionitio was used as the basis for a whole-day workshop on best practices in bioinformatics software development at the Australian Bioinformatics and Computational Biology Society (ABACBS) Annual Conference in November 2018 [48], delivered to an audience of 50 bioinformaticians from research and clinical institutes around Australia. In the first half of the workshop participants learnt how to set up a new software repository using Bionitio, allowing time for exploration of the codebase, discussion of key aspects of quality software, and an explanation of the processes that are automated by Bionitio. In the second half of the workshop participants learnt about test-driven development (TDD) and undertook an exercise to extend the codebase with new features, documentation, corresponding test cases, and linkage to revision control and continuous integration testing. In this setting, Bionitio's design as a simple-yet-realistic bioinformatics exemplar provides both a common codebase for coordination of workshop materials and an extensible platform for the delivery of hands-on practical activities. Additionally, by providing complete working examples in many different languages, Bionitio acts as a kind of "Rosetta Stone" and is therefore likely an excellent vehicle for comparative programming skills transfer.

Alignment with FAIR Principles and OSS Recommendations

In an effort to facilitate continued benefit from the digital assets related to data-intensive science, representatives from academia, industry, funding agencies, and publishers have proposed the FAIR Data Principles that aim to make experimental artefacts findable, accessible, interoperable and reusable for machines and people [49]. Jiménez *et al* have argued that poor development practices result in lower quality outputs that negatively impact reproducibility and reusability of research [50], and propose four principles for open source software development (OSS recommendations) that align well with the FAIR principles: 1) make source code publicly accessible from day one; 2) make software easy to discover by providing software metadata via a popular community registry; 3) adopt a licence and

comply with the licence of third-party dependencies; and 4) define clear and transparent contribution, governance and communication processes. Tools developed with Bionitio have a head start on satisfying both the FAIR principles and the first three OSS recommendations:

- they are publicly accessible in GitHub repositories with clearly indicated standard open source licences and user documentation;
- they are interoperable with other tools via standardised inputs and outputs and interfaces that follow long-established conventions;
- they are re-usable by virtue of the adoption of standard build procedures, the provision of clear documentation relating to installation and usage, containerisation with Docker, and integration into CWL;
- where appropriate, specific versions (with defined version numbers) can be made findable by the allocation of Digital Object Identifiers facilitated by Zenodo [51] through GitHub.

Importantly, Bionitio facilitates *compliance* with these principles, which is seen by Jiménez *et al* as the final (and, in our opinion, most difficult) step in organisational adoption.

Availability of supporting source code and requirements

- Project name: Bionitio
- Project home page: https://github.com/bionitio-team/bionitio
- Operating system(s): Any POSIX-like system.
- Programming language: Users can choose from: C, C++, C#, Clojure, Java, Javascript, Haskell, Perl, Python, R, Ruby, Rust
- Other requirements: BASH, curl and git are required for bionitio-boot.sh.

- License: MIT
- RRID: SCR_017259
- An archival copy of the code is available via the GigaScience database, GigaDB [52].

Declarations

Ethics approval and consent to participate: Not applicable

Consent for publication: Not applicable

Competing interests: The authors declare that they have no competing interests.

Funding: BP is supported by a Victorian Health and Medical Research Fellowship. HD is supported by an Australian Government Research Training Program (RTP) Scholarship, an Australian Genomics Health Alliance top up scholarship and a Murdoch Children's Research Institute top up scholarship. AL is supported by an Australian Government Research Training Program (RTP) Scholarship. PG is supported by an Australian Government Research Training Program (RTP) Scholarship.

Authors' contributions: TS, AL, HD and BP conceived of the project. All authors contributed to the design, implementation, testing and documentation of Bionitio. AS, CS, AL, HD, PG and BP contributed to manuscript drafting. All authors contributed to manuscript proofreading and final editing.

Acknowledgements: The authors would like to thank Melbourne Bioinformatics for providing computing resources for the development of Bionitio, and to the many users of the tool who have provided feedback about its use.

References

1. Baker M. 1,500 scientists lift the lid on reproducibility. Nature. 2016;533:452–4.

2. Wilson G. Software Carpentry: lessons learned. F1000Res. 2014;3:62.

3. Wilson G, Aruliah DA, Brown CT, Chue Hong NP, Davis M, Guy RT, et al. Best practices for scientific computing. PLoS Biol. 2014;12:e1001745.

4. Verma D, Gesell J, Siy H, Zand M. Lack of software engineering practices in the development of bioinformatics software. ICCGI. 2013;2013:57–62.

5. Segal J, Morris C. Developing Scientific Software. IEEE Softw. 2008;25:18–20.

6. Hannay JE, MacLeod C, Singer J, Langtangen HP, Pfahl D, Wilson G. How Do Scientists Develop and Use Scientific Software? Proceedings of the 2009 ICSE Workshop on Software Engineering for Computational Science and Engineering. Washington, DC, USA: IEEE Computer Society; 2009. p. 1–8.

7. Merali Z. Error: why scientific programming does not compute. Nature. 2010;467:775–7.

8. Joppa LN, McInerny G, Harper R, Salido L, Takeda K, O'Hara K, et al. Troubling Trends in Scientific Software Use. Science. American Association for the Advancement of Science; 2013;340:814–5.

9. Baxter SM, Day SW, Fetrow JS, Reisinger SJ. Scientific software development is not an oxymoron. PLoS Comput Biol. 2006;2:e87.

10. Lawlor B, Walsh P. Engineering bioinformatics: building reliability, performance and productivity into bioinformatics software. Bioengineered. 2015;6:193–203.

11. List M, Ebert P, Albrecht F. Ten Simple Rules for Developing Usable Software in Computational Biology. PLoS Comput Biol. 2017;13:e1005265.

12. Taschuk M, Wilson G. Ten simple rules for making research software more robust. PLoS Comput Biol. 2017;13:e1005412.

13. Prins P, de Ligt J, Tarasov A, Jansen RC, Cuppen E, Bourne PE. Toward effective software solutions for big biology. Nat Biotechnol. 2015;33:686–7.

14. Umarji M, Seaman C, Gunes Koru A, Liu H. Software Engineering Education for Bioinformatics [Internet]. 2009 22nd Conference on Software Engineering Education and Training. 2009. Available from: http://dx.doi.org/10.1109/cseet.2009.44

15. Howison J, Deelman E, McLennan MJ, Ferreira da Silva R, Herbsleb JD. Understanding the scientific software ecosystem and its impact: Current and future measures. Res Eval.; 2015;24:454–70.

16. Leprevost F da V, Barbosa VC, Francisco EL, Perez-Riverol Y, Carvalho PC. On best practices in the development of bioinformatics software. Front Genet. 2014;5:199.

17. Russell PH, Johnson RL, Ananthan S, Harnke B, Carlson NE. A large-scale analysis of bioinformatics code on GitHub. PLoS One. 2018;13:e0205898.

18. Seemann T. Ten recommendations for creating usable bioinformatics command line software. Gigascience. 2013;2:15.

19. Carey MA, Papin JA. Ten simple rules for biologists learning to program. PLoS Comput Biol. 2018;14:e1005871.

20. Huber W, Carey VJ, Gentleman R, Anders S, Carlson M, Carvalho BS, et al.

Orchestrating high-throughput genomic analysis with Bioconductor. Nat Methods. 2015;12:115–21.

21. Grüning B, Dale R, Sjödin A, Chapman BA, Rowe J, Tomkins-Tinch CH, et al. Bioconda: sustainable and comprehensive software distribution for the life sciences. Nat Methods. 2018;15:475–6.

22. Docker [Internet]. [cited 2019 Jul 8]. Available from: https://www.docker.com/

23. Amstutz P, Crusoe MR, Tijanić N, Chapman B, Chilton J, Heuer M, et al. Common Workflow Language, v1.0 [Internet]. 2016. Available from: https://doi.org/10.6084/m9.figshare.3115156.v2

24. Jackman SD, Mozgacheva T, Chen S, O'Huiginn B, Bailey L, Birol I, et al. ORCA: A Comprehensive Bioinformatics Container Environment for Education and Research. Bioinformatics. 2019

25. Belmann P, Dröge J, Bremges A, McHardy AC, Sczyrba A, Barton MD. Bioboxes: standardised containers for interchangeable bioinformatics software. GigaScience. 2015.

26. O'Connor BD, Yuen D, Chung V, Duncan AG, Liu XK, Patricia J, et al. The Dockstore: enabling modular, community-focused sharing of Docker-based genomics tools and workflows. F1000Res. 2017;6:52.

27. Glatard T, Kiar G, Aumentado-Armstrong T, Beck N, Bellec P, Bernard R, et al. Boutiques: a flexible framework to integrate command-line applications in computing platforms. Gigascience. 2018;7.

28. Crouch S, Hong NC, Hettrick S, Jackson M, Pawlik A, Sufi S, et al. The Software Sustainability Institute: Changing Research Software Attitudes and Practices. Comput Sci Eng. IEEE Computer Society; 2013;15:74–80.

29. Greenfeld AR. Cookiecutter [Internet]. Available from: https://github.com/audreyr/cookiecutter

30. Travis CI - Test and Deploy Your Code with Confidence [Internet]. [cited 2019 Mar 21]. Available from: https://travis-ci.org/

31. The Git Project. Git [Internet]. Git. [cited 2019 Apr 14]. Available from: https://git-scm.com/

32. IEEE and The Open Group. The Open Group Base Specifications Issue 7, 2018 edition [Internet]. 2018. Report No.: 1003.1-2008. Available from: http://pubs.opengroup.org/onlinepubs/9699919799/basedefs/V1_chap12.html

33. Free Software Foundation, Inc. GNU Coding Standards [Internet]. 2019 Feb. Available from: https://www.gnu.org/prep/standards/standards.html

34. Lee BD. Ten simple rules for documenting scientific software. PLoS Comput Biol. 2018;14:e1006561.

35. McIlroy MD, Pinson EN, Tague BA. UNIX Time-Sharing System: Foreword. Bell System Technical Journal. Wiley Online Library; 1978;57:1899–904.

36. Andrew H, David T. The Pragmatic Programmer: From Journeyman to Master. Addison Wesley Longman, Redwood City; 2000.

37. Cock PJA, Antao T, Chang JT, Chapman BA, Cox CJ, Dalke A, et al. Biopython: freely available Python tools for computational molecular biology and bioinformatics. Bioinformatics. 2009;25:1422–3.

38. Karimzadeh M, Hoffman MM. Top considerations for creating bioinformatics software documentation. Brief Bioinform. 2018;19:693–9.

39. Preston-Werner T. Semantic Versioning 2.0.0 [Internet]. [cited 2019 Mar 4]. Available from: https://semver.org/spec/v2.0.0.html

40. Raymond ES. The Art of UNIX Programming. Addison-Wesley Professional; 2003.

41. Gruening B, Sallou O, Moreno P, da Veiga Leprevost F, Ménager H, Søndergaard D, et al. Recommendations for the packaging and containerizing of bioinformatics software. F1000Res. 2019.

42. Choose an open source license [Internet]. [cited 2019 Mar 4]. Available from: https://choosealicense.com/

43. Johnson M. Building a Better ReadMe. Technical Communication. 1997;44:28–36.

44. Perez-Riverol Y, Gatto L, Wang R, Sachsenberg T, Uszkoreit J, Leprevost F da V, et al. Ten Simple Rules for Taking Advantage of Git and GitHub. PLoS Comput Biol. 2016;12:e1004947.

45. Glass RL. Facts and Fallacies of Software Engineering. Addison-Wesley Professional; 2003.

46. The Curl developers. Curl [Internet]. Curl: command line tool and library for transferring data with URLs. [cited 2019 Apr 14]. Available from: https://curl.haxx.se/

47. Tractenberg RE, Lindvall JM, Attwood TK, Via A. The Mastery Rubric for Bioinformatics: supporting design and evaluation of career-spanning education and training. bioRxiv [Internet]. biorxiv.org; 2019; Available from: https://www.biorxiv.org/content/10.1101/655456v1.abstract

48. ABACBS-2018 Annual Conference [Internet]. [cited 18 June, 2019]. Available from: https://www.abacbs.org/conference2018/about

49. Wilkinson MD, Dumontier M, Aalbersberg IJJ, Appleton G, Axton M, Baak A, et al. The FAIR Guiding Principles for scientific data management and stewardship. Sci Data. 2016;3:160018.

50. Jiménez RC, Kuzak M, Alhamdoosh M, Barker M, Batut B, Borg M, et al. Four simple recommendations to encourage best practices in research software. F1000Res. 2017;6.

51. Zenodo [Internet]. [cited 18 June, 2019]. Available from: https://zenodo.org/

52. Georgeson P, Syme A, Sloggett C, Chung J, Dashnow H, Milton M, et al. Supporting data for “Bionitio: demonstrating and facilitating best practices for bioinformatics command-line software” GigaScience Database. 2019. Available from: http://dx.doi.org/10.5524/100640

Additional Files

File name: Additional file 1

File format: Microsoft Word

Title of data: Table 1

Description of data: Contents of Table 1 to be included in the manuscript where indicated.

Table Click here to access/download;Table;Additional file 1.docx

language	build/deploy	FASTA reading	command line argument parsing	unit testing	logging	static analysis	code format
C	make	kseq	getopt	assert	custom	lint	clang-format
C++	cmake	Seqan	boost::program_options	catch	boost::log	cppcheck	clang-format
C#	dotnet	.Net Bio	Microsoft.Extensions.CommandLineUtils	Microsoft.VisualStudio.TestTools.UnitTesting	Serilog	N/A	N/A
Clojure	lieningen	Bioclojure	clojure.tools.cli	clojure.test	timbre	Eastwood	cljfmt
Java	maven	biojava	Apache Commons	junit	custom	checkstyle	checkstyle
Javascript	node	fasta-parser	commander	mocha	winston	N/A	standard
Haskell	stack	BioHaskell	optparse-applicative	hspec	hslogger	hlint	N/A
Perl	N/A	BioPerl	Getopt::ArgParese	Test::More	Log::Log4perl	perlcritic	perltidy
Python	pip	biopython	argparse	unittest	logging	pylint	N/A
R	R	seqinr	optparse	testthat	logging	lintr	N/A
Ruby	gem	bioruby	optparse	Test::Unit	logger	N/A	N/A
Rust	cargo	bio::io::fasta	argparse	native test feature of Rust	log, log4rs	N/A	rustfmt

Table 1. Standard libraries and tools employed by each implementation of Bionitio. Instances where no appropriate option was available are marked with N/A.

Figure

Click here to access/download;Figure;figure1.png

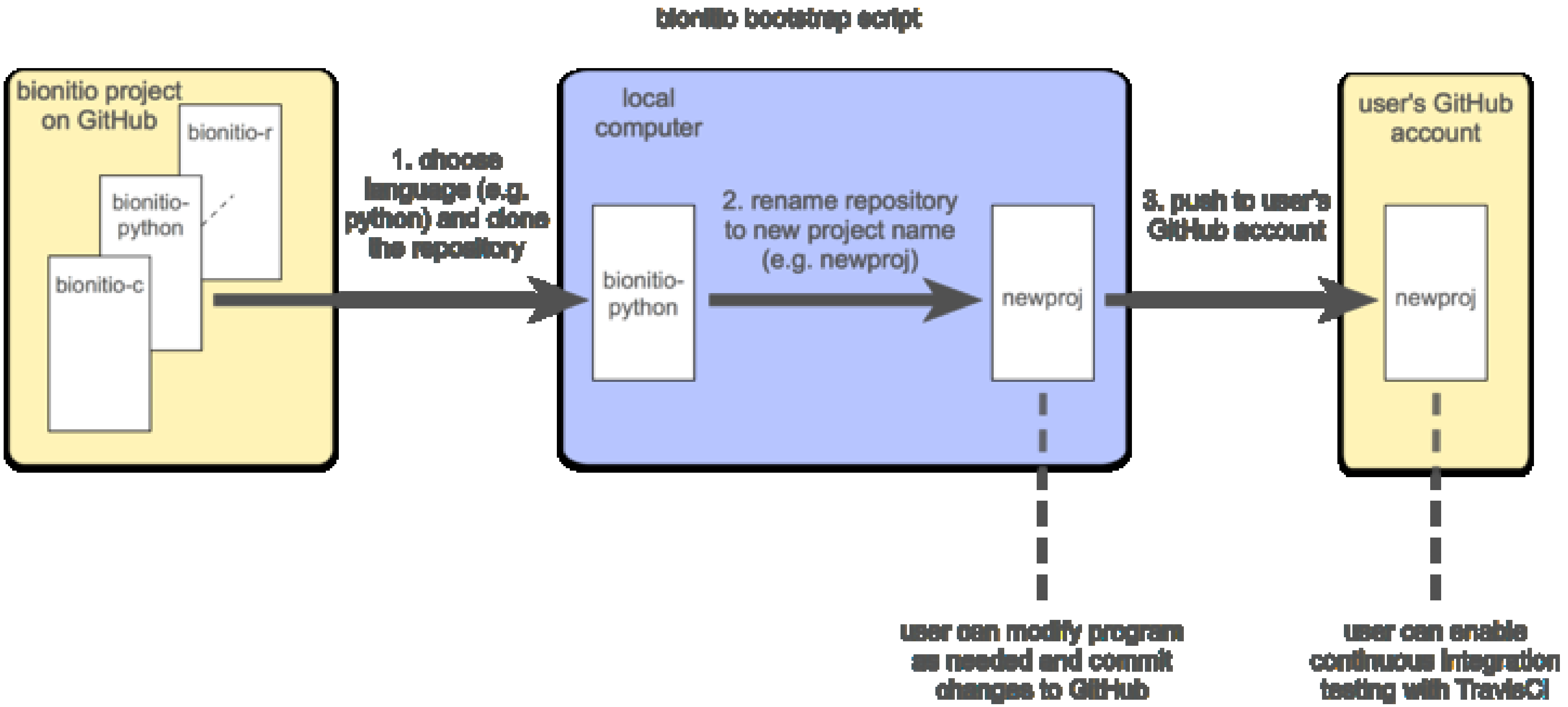

SVG version of figure1.png

Click here to access/download
Supplementary Material
figure1.svg
