# Supplementary material for: Bionitio: demonstrating and facilitating best practices for bioinformatics command-line software
Source: Gigascience. 2019 Sep 23;8(9):giz109. doi: 10.1093/gigascience/giz109 (PMC6755254; doi:10.1093/gigascience/giz109)
Supplement: giz109_Response_to_Reviewer_Comments_Original_Submission [file giz109_response_to_reviewer_comments_original_submission.pdf]

Author's Response To Reviewer Comments

Close

16 July 2019

To the Editor-in-Chief and Executive Editor, GigaScience,

We thank the Editor and Reviewers for their insightful and constructive comments on our submission "Bionitio: demonstrating and facilitating best practices for bioinformatics command-line software" (GIGA-D-19-00145). In the following document we respond to each of the reviewer comments and say what has changed in the software and text to address each point. In the revised manuscript we have used red font to indicate changes that we have made to the body of the text. We believe that the suggested changes have significantly improved the quality of the paper and the corresponding Bionitio tool.

The responses here are presented in the order that the comments appear in the manuscript review.

Editor Comments

The reviewers agree that the tool itself is a useful contribution, overall. However, they also have some constructive suggestions for improving the manuscript.

In particular, I agree with reviewer 1 that, ideally, the manuscript should also present "an evidence-backed testimony about the tool's efficacy in correcting the problems stated in the introduction."
I understand that a typical, quantitative benchmarking exercise may not be possible for this type of tool, but reviewer 1 has some good pointers regarding issues that should be discussed in more detail (for example, regarding FAIR principles and how your approach suggested in the paper can help in this regard).

Reviewer 2 has some notes on installation and running the tool that may give you some hints for minor improvements or corrections.

The reviewers also suggest to provide the tool via a container (e.g. docker), especially as it is meant to be helpful for beginners.

In addition, please register any new software application in the SciCrunch.org database to receive a RRID (Research Resource Identification Initiative ID) number, and include this in your manuscript. This will facilitate tracking, reproducibility and re-use of your tool.

Response

We concur with the Editor that a qualitative benchmarking exercise is challenging for this type of tool, and that a detailed discussion of our alignment with FAIR principles is a valuable contribution to the paper. In light of these remarks we have included a section on how Bionitio enables bioinformaticians to easily adopt many of the key FAIR principles and have additionally linked this to recent work on related recommendations for Open Source research software. Disclaimer: a Bionitio author (B Pope) a co-author on the latter recommendations.

We have attempted to address issues related to installation and running the tool outlined by Reviewer 2 and have provided Docker containers for each of the Bionitio implementations, as well as the bootstrap script, and updated the documentation accordingly.

We have applied for registration of Bionitio through SciCrunch.org and received an RRID of SCR_017259. We have included this in the manuscript.

Reviewer 1

Comment 1 (and comment 12)

The limitation of this manuscript, in my mind, is mostly that it reads like more of an instruction manual and list of general best practices than a detailed technical write up about the contribution made, and an evidence-backed testimony about its efficacy in correcting the problems stated in the introduction.

Response

We believe that one of the contributions of Bionitio is that it provides a consolidation of many disparate sources of best practices for software development in bioinformatics. Indeed, the features present in Bionitio are distilled from more than 25 different partially overlapping recommendations. We also believe that it is a contribution of our manuscript to explicitly link those recommendations to the features present in our tool. Therefore, there is necessity to list our sources and argue for their significance. Another key contribution of our paper is to show how easily a new project can be created with our tool, as a step-by-step guide to its main features.

However, we also agree that our manuscript could have made our contributions clearer and argued further for its efficacy in correcting the problems stated in the introduction.

We also agree with the Editor that "a typical, quantitative benchmarking exercise may not be possible for this type of tool".

In light of these comments we have made considerable changes to the manuscript.

The following text was added to the conclusion to show how Bionitio helps users to adopt FAIR principles and related recommendations for open source software:

Alignment with FAIR Principles and OSS Recommendations

In an effort to facilitate continued benefit from the digital assets related to data-intensive science, representatives from academia, industry, funding agencies, and publishers have proposed the FAIR Data Principles that aim to make experimental artefacts findable, accessible, interoperable and reusable for machines and people [48]. Jiménez et al have argued that poor development practices result in lower quality outputs that negatively impact reproducibility and reusability of research [49], and propose four principles for open source software development (OSS recommendations) that align well with the FAIR principles: 1) make source code publicly accessible from day one; 2) make software easy to discover by providing software metadata via a popular community registry; 3) adopt a licence and comply with the licence of third-party dependencies; and 4) define clear and transparent contribution, governance and communication processes. Tools developed with Bionitio have a head start on satisfying both the FAIR principles and the first three OSS recommendations:

- they are publicly accessible in GitHub repositories with clearly indicated standard open source licences and user documentation;
- they are interoperable with other tools via standardised inputs and outputs and interfaces that follow long-established conventions;
- they are re-usable by virtue of the adoption of standard build procedures, the provision of clear documentation relating to installation and usage, containerisation with Docker, and integration into CWL;
- where appropriate, specific versions (with defined version numbers) can be made findable by the allocation of Digital Object Identifiers facilitated by Zenodo [50] through GitHub.

Importantly, Bionitio facilitates compliance with these principles, which is seen by Jiménez et al as the final (and, in our opinion, most difficult) step in organisational adoption.

The following text was added to the conclusion to outline Bionitio's role in education and training by relating it to the Mastery Rubric for Bioinformatics proposed by Tractenberg et al, along with our own experience in using it to deliver a national bioinformatics workshop (in Australia):

Role in education and training

In very recent work Tractenberg et al have developed a Mastery Rubric for Bioinformatics with the goal of better defining skills development and competencies in the discipline [46]. In this framework,

competency in computational methods ranges through five levels, from novice (stage 1) to independent bioinformatics practitioner (stage 5). One of the goals of Bionitio is to support education and training for advancing bioinformaticians from stage 3 - learning best practices in programming and writing basic code - to stage 5 - developing new software that is useful, efficient, standardized, well-documented and reproducible. As an example of this application, Bionitio was used as the basis for a whole-day workshop on best practices in bioinformatics software development at the Australian Bioinformatics and Computational Biology Society (ABACBS) Annual Conference in November 2018 [47], delivered to an audience of 50 bioinformaticians from research and clinical institutes around Australia. In the first half of the workshop participants learnt how to set up a new software repository using Bionitio, allowing time for exploration of the codebase, discussion of key aspects of quality software, and an explanation of the processes that are automated by Bionitio. In the second half of the workshop participants learnt about test-driven development (TDD) and undertook an exercise to extend the codebase with new features, documentation, corresponding test cases, and linkage to revision control and continuous integration testing. In this setting, Bionitio's design as a simple-yet-realistic bioinformatics exemplar provides both a common codebase for coordination of workshop materials and an extensible platform for the delivery of hands-on practical activities. Additionally, by providing complete working examples in many different languages, Bionitio acts as a kind of "Rosetta Stone" and is therefore likely an excellent vehicle for comparative programming skills transfer.

We have also expanded the third paragraph in the Conclusions to emphasise why we think Bionitio is a significant contribution on top of the already existing recommendations in the literature (and the main motivation for its creation):

The challenges faced by the bioinformatics and science communities in building better quality software are well known, and there is no shortage of practical recommendations to be found in the literature. These guidelines are undoubtedly useful to beginners, however we believe they fall short in two ways. First, they are spread over multiple manuscripts that only partially overlap in their recommendations, therefore some level of consolidation is needed. Second, they are static artefacts that point to good practices but do not remove the considerable burden of applying them in real code. These two observations motivated the creation of Bionitio, both as a way of collecting commonly recommended best practices, and as a way of demonstrating and facilitating their use. Therefore, a significant contribution of our work is to build a tool that can both illustrate best practices by example but also make it easy to use them in new projects. In this sense Bionitio takes a much more active role in the dissemination and compliance with these principles.

We have also emphasised the contribution that this tool makes to improving software development in bioinformatics as per comments 13,14,15 and 21 below.

Comment 2

(section 1; paragraph 2) How is "correctness" evaluated in your mind? In research truth is often unknown by definition, so perhaps choose a less loaded word or elaborate on how this is evaluated.

Response

We agree with the reviewer that truth can be elusive in science, and therefore by correctness we mean that the software implements its intended functionality; so it is correct in the sense that it meets its specification (whether that specification be formally defined, or, more likely, part of the informal intentions that are known to the author(s)). Following the advice of the reviewer we have used a less loaded way to describe this, and changed the manuscript as follows:

Given the results-driven nature of research, the functional aspects of scientific programs (e.g. correctness whether expected inputs produce expected outputs) are heavily emphasised at the expense of the non-functional ones (e.g. usability, maintainability, interoperability, efficiency).

Comment 3

Duplicate heading at start of paper? Both "Findings" and "Background"

Response

We believe that this formatting follows the suggested GigaScience Technical Note style

(https://academic.oup.com/gigascience/pages/technical_note), where in the main text, "Findings" is a larger heading including the subheadings Background, Implementation, Methods, Conclusions, etc. If our interpretation of the formatting guidelines is incorrect, we are confident that this can be fixed in the final proof.

Comment 4

(section 1; paragraph 2, last sentence) Some "specifications" or recommendations, such as Nature Publishing's software checklist, and some 10-simple-rules articles in pnas related to scientific software. Are these the types of things you're referring to? If so, might be worth mentioning how they can exist but perhaps are harder to define for a specific (quickly moving) domain beyond the "basics".

Response

In this part we are referring to "software requirements specifications" that are commonly used in Software Engineering to define the functional and non-functional requirements of software being developed. We have changed the text to "software requirements specifications (SRSs)" to clarify this point.

Comment 5

(section 1; paragraph 4) abovementioned -> above-mentioned

Response

Corrected.

Comment 6

(section 1; second-last paragraph) "more likely to adopt good practices" <- have you witnessed this in the wild with bionitio, yet? I agree that in principle I'd expect this result, but giving students or researchers the tool and saying nothing else, then coming back at the end of the process, is this the outcome we get? The biggest places I see this not continuing beyond the boilerplate is documentation and testing. This could potentially also be answered if Cookiecutter has successes that you could reference.

Response

We agree with the reviewer that this is an expected result, however we have not formally tested it, and, for now it sits here as a hypothesis. We have reworded the sentence to make this point clearer:

The key point is that they are building on solid foundations, and because a lot of the mundane-but-important boilerplate is provided by Bionitio, there are fewer barriers to adopting good practices from the start.

Comment 7

(command line argument parsing) have you considered integrating these command-line descriptions with standard tools for shipping workflows to C(G)PUs, like Common Workflow Language (commonwl.org), Boutiques (boutiques.github.io), or others? It would be an additional feature you could add on top of each language-specific implementation that would make not only consuming the tools even more uniform, but enable scaling them out for large datasets more accessible for developers.

Response

We agree with the reviewer that this would be a useful additional feature, and therefore have added example CWL tool wrappers for each implementation of Bionitio. This addition was greatly facilitated by the fact that each Bionitio implementation has the same command line interface, and (now) comes with a Docker container. We have updated the online documentation for Bionitio to include information about this, and have made the following changes to the manuscript:

In the Background section:

Operating system virtualisation services, such as Docker [22], and workflow specification languages, such as the Common Workflow Language (CWL) [23], have improved portability and reproducibility of tools and pipelines [12,24–26].
...

Specifically, every new Bionitio-created project includes … containerisation with Docker, and a CWL wrapper.

In the Design and Implementation section:

CWL tool wrapper

Bioinformatics pipelines — where multiple tools are chained together to perform an overall analysis — create further challenges for reproducible science. This has motivated the creation of pipeline frameworks that allow the logic of such computations to be abstracted from the details of how they are executed. An emerging standard in this area is the Common Workflow Language (CWL) that is supported by several popular workflow engines. CWL comprises two declarative sub-languages: workflow descriptions, that define data flow patterns between pipeline stages; and command line tool descriptions, that define the interfaces of tools in a platform independent manner. Each Bionitio implementation provides a CWL tool description "bionitio.cwl", that facilitates its incorporation into CWL pipelines, and takes advantage of CWL's support for invoking programs within Docker containers.

We have also updated the README.md files for each implementation of Bionitio to include information about how to use the CWL tool wrapper and included running the CWL tool wrapper within Travis CI testing.

Comment 8

(software packaging) there is also no mention of virtualization/containerization here, such as Docker or Singularity, that would also increase the portability of these packages. Have the authors considered this to further minimize this issue?

Response

We agree with the reviewer that this would be a useful additional feature, and therefore have added example Docker container definitions for each implementation of Bionitio, and also the bootstrap script. We have made the following changes to the manuscript:

In the Abstract:

Key features include … , and containerisation.

In the Background section:

Specifically, every new Bionitio-created project includes … containerisation with Docker, and a CWL wrapper.

In the Design and Implementation section:

Sub-heading changed from "Standardised software packaging using programming language specific mechanisms" to "Standardised software packaging and containerisation".

Text added:

Standard packaging also helps with containerisation, which is becoming increasingly useful in bioinformatics [40]. Docker containers are a popular implementation of this concept, where the underlying operating system is virtualised and packaged alongside tools and their dependencies. This makes it easy to install "containerised" software on any platform that supports Docker, and facilitates reproducibility by enabling the exact same software build to be used on every system. Each Bionitio implementation comes with a "Dockerfile" that encodes all the necessary information needed to create a

containerised version of the tool. As an added benefit, the Docker container is used in Travis Continuous Integration testing, which both simplifies the use of Travis and also enables the functionality of the container itself to be included in the tests.

In the Methods section we added the following text:

Alternatively, the bootstrap script can be run from a Docker container published on DockerHub (https://cloud.docker.com/u/bionitio/repository/docker/bionitio/bionitio-boot):

```
$ docker run -it -v "$(pwd):/out" --rm bionitio/bionitio-boot \
-i python -n newproj -c BSD-3-Clause
```

Comment 9

(methods; choosing a language) do you have any way to recommend language selection for users? If they're truly new to all of these, maybe coming from a MATLAB background like many who learned to program through coursework, what guidance does Bionitio provide here? Is Python a general default, or just for this example? If it is, where is that justified? The caveat with providing 12 options is that a bit of hand holding may be required to guide the choice for much of your target audience.

Response

We agree with the reviewer that choice of programming language can be difficult for absolute beginners. It is difficult to get empirical evidence to support any language default (and for this reason Bionitio does not have a default language). However, the selection of implementation languages chosen was guided by the results reported in [13]. From an analysis of 1,720 bioinformatics repositories on GitHub they observed: "The main dataset contained a greater proportion of code written in interpreted or hybrid interpreted/compiled (such as Python) and dynamically typed languages" and "Our data support the intuition that Java, Python and R are more succinct than lower-level languages such as C and C++" Taking these observations together, Python appears to be reasonable starting language for beginners. To assist beginners with their choice of language we have updated the README (https://github.com/bionitio-team/bionitio) documentation for Bionitio to include:

If you are new to programming, and do not know which programming language to use, then we recommend picking one of the high-level interpreted languages that are popular in Bioinformatics, such as Python or R. You may also need to seek advice from your peers about which language(s) are most appropriate for your purposes. We have tried to cover as many popular languages as possible, and apologise if your preference is not currently available. However, we also welcome new implementations of Bionitio in languages not already covered.

We have also added the following text to the manuscript:

For users relatively new to programming, with no prior constraints on their choice of language, we recommend they choose a high-level interpreted language such as Python or R.

Comment 10

can you justify the claims about it being an "excellent vehicle for education"? Any sort of case study or example from similar tools being effective, etc…

Response

We believe that Bionitio is fairly unique in its approach to templating best practices in Bioinformatics software development, and therefore it is unlikely that such an approach has been formally studied in the context of education practices, and unfortunately we are not aware of such resources (even beyond bioinformatics). However, as mentioned in our cover letter, we have used Bionitio as the basis for a popular (whole day) workshop hosted at the Australian Bioinformatics and Computational Biology Society (ABACBS) annual conference in 2018 (https://www.abacbs.org/conference2018) with ~50 paying attendees from around the country. We conducted a survey of the attendees to assess the quality and utility of the workshop. In response to the question "This was a useful workshop that enhanced my knowledge and skills" out of 18 respondents 94.44% agreed or strongly agreed. Given the

success of the initial workshop, we ran another in May 2019, with 14 attendees. From formal feedback received from the second workshop, in response to the question "My overall impression is that this is a useful workshop that enhanced my knowledge and skills" we received a score of 4.8/5 from 11 respondents. We appreciate that this is anecdotal evidence and is not supported by a rigorous experiment and therefore we have not discussed the workshop feedback in the manuscript. However, we have reduced the strength of our claim in the manuscript by adding a qualifier:

Additionally, by providing complete working examples in many different languages, Bionitio acts as a kind of "Rosetta Stone" and is therefore likely an excellent vehicle for comparative programming skills transfer.

We have also addressed Bionitio's role in training and education more thoroughly in the Conclusion as mentioned in our response to Comment 1 above.

Comment 11

figure 1 text is barely readable, and boxes are odd relative sizes with a fair amount of wasted foreground (coloured) space. Colour doesn't seem to convey much information. I didn't find this figure particularly useful or instructive. I.e. I don't know any better how I would use bionitio, or what exactly it'll create (just that it draws from a boiler plate). Maybe repurpose this figure to be more of a "schematic" of what is contained within a bionitio-created-project (is there a more concise name for these?), and then a more streamlined version of what is currently here.

Response

We included this figure in the manuscript because it serves to visually represent how a new project is started by the bootstrap script. We have found that this has been a particular issue of confusion for new users, especially those who are unfamiliar with Git and GitHub. The colours represent the location of code, either in GitHub (yellow) or the local machine (purple). To streamline this figure, we have removed the grey background, replaced the external arrows with dashed lines. We will submit a high-resolution version in our resubmission, and include a resolut
