# Supplementary material for: Bionitio: demonstrating and facilitating best practices for bioinformatics command-line software
Source: Gigascience. 2019 Sep 23;8(9):giz109. doi: 10.1093/gigascience/giz109 (PMC6755254; doi:10.1093/gigascience/giz109)
Supplement: giz109_Response_to_Reviewer_Comments_Revision_1 [file giz109_response_to_reviewer_comments_revision_1.pdf]

Author's Response To Reviewer Comments

Close

Dear GigaScience Editors,

Thank you for accepting our manuscript for publication.

In response to the comments from the Editor and reviewer we have made the following small changes to the manuscript:

1) We have cited the Boutiques software, as recommended by the reviewer.
2) We have added a link to the archival snapshot of your code in gigaDB.
3) We have made minor corrections for typos, grammar, format, and have removed any tracking and red color that was made to highlight changes for the reviewers.

Kind regards,
Bernard Pope

Close
