# Supplementary material for: Bionitio: demonstrating and facilitating best practices for bioinformatics command-line software
Source: Gigascience. 2019 Sep 23;8(9):giz109. doi: 10.1093/gigascience/giz109 (PMC6755254; doi:10.1093/gigascience/giz109)
Supplement: giz109_Reviewer_1_Report_Original_Submission — Gregory Kiar -- 5/19/2019 Reviewed [file giz109_reviewer_1_report_original_submission.pdf]

Reviewer Report

Title: Bionitio: demonstrating and facilitating best practices for bioinformatics command-line software

Version: Original Submission **Date:** 5/19/2019

Reviewer name: Gregory Kiar

Reviewer Comments to Author:

(Review) Bionitio: demonstrating and facilitating best practices for bioinformatics command-line software
Greg Kiar
May 19th, 2019
In this manuscript the authors present Bionitio, a tool which lowers the barrier for adopting best practices in bioinformatics-focused scientific tool development. The authors discuss the issues with current practices in software development, and propose this command-line utility for generating "skeleton" packages to be used as scaffolding for future tool development and example implementations of their proposed best practices, including but not limited to packaging, testing, versioning, and informative error handling. In general the manuscript is very well written, clear, and is introducing a tool that provides clear value to the community. The limitation of this manuscript, in my mind, is mostly that it reads like more of an instruction manual and list of general best practices than a detailed technical write up about the contribution made, and an evidence-backed testimony about its efficacy in correcting the problems stated in the introduction.
Minor comments:
- Duplicate heading at start of paper? Both "Findings" and "Background"
- (section 1; paragraph 2) How is "correctness" evaluated in your mind? In research truth is often unknown by definition, so perhaps choose a less loaded word or elaborate on how this is evaluated.
- (section 1; paragraph 2, last sentence) Some "specifications" or recommendations, such as Nature Publishing's software checklist, and some 10-simple-rules articles in pnas related to scientific software. Are these the types of things you're referring to? If so, might be worth mentioning how they can exist but perhaps are harder to define for a specific (quickly moving) domain beyond the "basics".
- (section 1; paragraph 4) abovementioned -> above-mentioned
- (section 1; second-last paragraph) "more likely to adopt good practices" <- have you witnessed this in the wild with bionitio, yet? I agree that in principle I'd expect this result, but giving students or researchers the tool and saying nothing else, then coming back at the end of the process, is this the outcome we get? The biggest places I see this not continuing beyond the boilerplate is documentation and testing. This could potentially also be answered if Cookiecutter has successes that you could reference.
- (command line argument parsing) have you considered integrating these command-line descriptions with standard tools for shipping workflows to C(G)PUs, like Common Workflow Language (commonwl.org), Boutiques (boutiques.github.io), or others? It would be an additional feature you could add on top of each language-specific implementation that would make not only consuming the tools

even more uniform, but enable scaling them out for large datasets more accessible for developers.
- (software packaging) there is also no mention of virtualization/containerization here, such as Docker or Singularity, that would also increase the portability of these packages. Have the authors considered this to further minimize this issue?
- (methods; choosing a language) do you have any way to recommend language selection for users? If they're truly new to all of these, maybe coming from a MATLAB background like many who learned to program through coursework, what guidance does Bionitio provide here? Is Python a general default, or just for this example? If it is, where is that justified? The caveat with providing 12 options is that a bit of hand holding may be required to guide the choice for much of your target audience.
- (conclusion) can you justify the claims about it being an "excellent vehicle for education"? Any sort of case study or example from similar tools being effective, etc...
Significant comments:
- figure 1 text is barely readable, and boxes are odd relative sizes with a fair amount of wasted foreground (coloured) space. Colour doesn't seem to convey much information. I didn't find this figure particularly useful or instructive. I.e. I don't know any better how I would use bionitio, or what exactly it'll create (just that it draws from a boiler plate). Maybe repurpose this figure to be more of a "schematic" of what is contained within a bionitio-created-project (is there a more concise name for these?), and then a more streamlined version of what is currently here.
- I felt that while the manuscript introduces a tool which is certainly of use to a community of scientific software developers, the focus of the paper is more based on the justification of which components are included in this tool, rather than the technical nature or efficacy of the contribution. With guides that exist and "best practices" that were even mentioned in the 10-simple-rules article, I believe the article would benefit from significant rewriting to be focused on the contributions of these authors and their tool, rather than an extended summary of what are commonly accepted as best practices for software development. While I acknowledge the novel and valuable contribution presented in this paper, I feel the manuscript does not highlight this contribution adequately.
- One concern I have with making it easier for people to continue making their own tools is exactly to the point mentioned at one point in the manuscript, of "never repeat yourself." In my area of research, computational neuroscience, essentially every pipeline has been built handfuls of times, and the answers aren't particularly replicable across implementations. This of course raises a whole other set of issues in terms of the quality of software being produced, because even if we encourage developers to adopt existing tools where possible, if they don't adopt the same ones for the same tasks, how can we meaningfully compare their implementations? This ties in to the FAIR principles, which I was surprised not to see mention of in this work, as they are closely aligned with the aim of bionitio to my understanding. The missing piece in bionitio, of course, would then be that of publishing tools and ensuring the findability of software that people will make. This of course doesn't solve the issue, but at least enables the easier evaluation of various implementations towards the same end. As mentioned above, the Boutiques initiative (disclaimer: I am a co-lead on this project) makes efforts to make sure tools, once they exist, are able to be shared/consumed FAIR-ly, so could potentially be referenced in a discussion on this point. The paper of this tool is on Gigascience (https://doi.org/10.1093/gigascience/giy016) and a recent poster focusing on FAIR software workflows can be found here: https://doi.org/10.6084/m9.figshare.8143241.v2 . I would appreciate if the authors

discussed this point, the obvious risk that their tool introduces into the field by virtue of increasing the accessibility of tool development, and how they propose their contribution is either worth this added risk or how they intend on enabling the evaluation of it.

Level of Interest

Please indicate how interesting you found the manuscript: Choose an item.

Quality of Written English

Please indicate the quality of language in the manuscript: Choose an item.

Declaration of Competing Interests

Please complete a declaration of competing interests, considering the following questions:

- Have you in the past five years received reimbursements, fees, funding, or salary from an organisation that may in any way gain or lose financially from the publication of this manuscript, either now or in the future?
- Do you hold any stocks or shares in an organisation that may in any way gain or lose financially from the publication of this manuscript, either now or in the future?
- Do you hold or are you currently applying for any patents relating to the content of the manuscript?
- Have you received reimbursements, fees, funding, or salary from an organization that holds or has applied for patents relating to the content of the manuscript?
- Do you have any other financial competing interests?
- Do you have any non-financial competing interests in relation to this paper?

If you can answer no to all of the above, write 'I declare that I have no competing interests' below. If your reply is yes to any, please give details below.

I declare that I have no competing interests

I agree to the open peer review policy of the journal. I understand that my name will be included on my report to the authors and, if the manuscript is accepted for publication, my named report including any attachments I upload will be posted on the website along with the authors' responses. I agree for my report to be made available under an Open Access Creative Commons CC-BY license (http://creativecommons.org/licenses/by/4.0/). I understand that any comments which I do not wish to be included in my named report can be included as confidential comments to the editors, which will not be published.

Choose an item.

To further support our reviewers, we have joined with Publons, where you can gain additional credit to further highlight your hard work (see: https://publons.com/journal/530/gigascience). On publication of this paper, your review will be automatically added to Publons, you can then choose whether or not to claim your Publons credit. I understand this statement.

Yes Choose an item.
