# Supplementary material for: Bionitio: demonstrating and facilitating best practices for bioinformatics command-line software
Source: Gigascience. 2019 Sep 23;8(9):giz109. doi: 10.1093/gigascience/giz109 (PMC6755254; doi:10.1093/gigascience/giz109)
Supplement: giz109_Reviewer_2_Report_Original_Submission — Lars Ailo Bongo -- 5/20/2019 Reviewed [file giz109_reviewer_2_report_original_submission.pdf]

Reviewer Report

Title: Bionitio: demonstrating and facilitating best practices for bioinformatics command-line software

Version: Original Submission **Date:** 5/20/2019

Reviewer name: Lars Ailo Bongo

Reviewer Comments to Author:

The paper addresses an important challenge in bioinformatics; how to improve code quality, including documentation and testing. The proposed approach, bionitio, are templates that create a code repository for many popular (and perhaps not so popular) programming languages in bioinformatics. The tool can also add the repository to the user's GitHub. The paper describes and motivates best practices for software development, by for example using unit tests.
I believe the paper is very useful to read for inexperienced software developers since it explains many best practices for writing usable tools. The templates then show how these are implemented. However, I wonder if the command line is the best starting point for inexperienced programmers? And if not starting with an IDE that provides similar templates would be better? The paper could therefore discuss how bionitio can be used from an IDE. I also wonder if the team plan to provide bionitio templates for some popular IDEs? Finally, are there some numbers that can document how much bionitio is used, and who the users are? Perhaps something from GitHub?
The GitHub repository for bionitio has 563 commits, 11 forks, and 19 stars. The code seems to be in active development. The last commit was about a month ago. It has nice documentation, and an issue tracker.
I tested bionitio-python in Ubuntu on Windows. It has Python2 as default, so by following the instructions I could install the code (with Python2), but not run it. However, by installing the code with pip3 I could use it as described in the documentation. But this solution may not be obvious for beginner programmers, so the documentation should take this into account. I also found that the example program treated invalid fasta files (for example setup.py) as empty fasta files. I believe according to the documentation this should have resulted in an "invalid file" exit value instead of success (zero).
I also asked three members of my team to test it (Dr. Einar HolsbÃ¸, Morten GrÃ¸nnesby, and Jo Inge Arnes). All are experienced programmers. They tested bionitio for R, Python, C++, and C#.
We cloned the C#-repository and opened the included solution in Visual Studio 2017. In the debugger options, we gave the path to our FASTA-file as a command-line parameter. We placed a break-point at the last return statement in the main function to prevent the application from exiting before we could read the results. The application was launched using the debugger, and the outputted results were as expected. Next, we generated a boiler-plate project for C#. Since there were no available bat-scritps in the bionitio-repository, we used PowerShell to run the boot/bionitio-boot.sh script.
In the documentation, C# was not mentioned as one of the available languages. We tried to use -i csharp as a parameter, which was recognized by the script. The C# project files were generated and the solution worked. The integration with GitHub, however, did not work. When we ran the script, a different command-line window was opened, asking for a GitHub password. When typing in the password, it was

shown in clear text. Nothing happened when hitting the Enter-key. The GitHub-repository was not created.
The C++ version requires a bit more manual configuration, since the `CMake`/`Make` install does not copy the built executable to a standard location (such as usr/local/bin). It also depends on the `seqan` library that has to be downloaded separately.

Level of Interest

Please indicate how interesting you found the manuscript: Choose an item.

Quality of Written English

Please indicate the quality of language in the manuscript: Choose an item.

Declaration of Competing Interests

Please complete a declaration of competing interests, considering the following questions:

- Have you in the past five years received reimbursements, fees, funding, or salary from an organisation that may in any way gain or lose financially from the publication of this manuscript, either now or in the future?
- Do you hold any stocks or shares in an organisation that may in any way gain or lose financially from the publication of this manuscript, either now or in the future?
- Do you hold or are you currently applying for any patents relating to the content of the manuscript?
- Have you received reimbursements, fees, funding, or salary from an organization that holds or has applied for patents relating to the content of the manuscript?
- Do you have any other financial competing interests?
- Do you have any non-financial competing interests in relation to this paper?

If you can answer no to all of the above, write 'I declare that I have no competing interests' below. If your reply is yes to any, please give details below.

I declare that I have no competing interests

I agree to the open peer review policy of the journal. I understand that my name will be included on my report to the authors and, if the manuscript is accepted for publication, my named report including any attachments I upload will be posted on the website along with the authors' responses. I agree for my report to be made available under an Open Access Creative Commons CC-BY license (http://creativecommons.org/licenses/by/4.0/). I understand that any comments which I do not wish to be included in my named report can be included as confidential comments to the editors, which will not be published.

Choose an item.

To further support our reviewers, we have joined with Publons, where you can gain additional credit to further highlight your hard work (see: https://publons.com/journal/530/gigascience). On publication of this paper, your review will be automatically added to Publons, you can then choose whether or not to claim your Publons credit. I understand this statement.

Yes Choose an item.
